# Nickel-Free Anodes for Hydrocarbon- and Ammonia-Fueled Solid Oxide Fuel Cells: A Review

**DOI:** 10.3390/ma19214731

**Published:** 2026-11-06

**Authors:** Nur Hanisah Hadi, Mustafa Anwar, Yathavan Subramanian, Nursultan Aidarbekov, Kenzhebatyr Bekmyrza, Packiaraj Rajagopal, Asset Kabyshev, Muhammed Ali Shaikh Abdul Kader Abdul Hameed

**Affiliations:** 1 Fuel Cell Institute, Universiti Kebangsaan Malaysia, Bangi 43600, Selangor, Malaysia; nisahhadi31@gmail.com; 2 Department of Energy Systems Engineering, U.S.-Pakistan Centre for Advanced Studies in Energy (USPCAS-E), National University of Sciences & Technology (NUST), H-12 Sector, Islamabad 44000, Pakistan; mustafa@uspcase.nust.edu.pk; 3 Department of Chemical and Environmental Engineering, Universidad Técnica Federico Santa María, Vicuña Mackenna 3939, Santiago 8940897, Chile; 4 Institute of Physical and Technical Sciences, L.N. Gumilyov Eurasian National University, Astana 010008, Kazakhstan; aidarbekov_nk_1@enu.kz (N.A.); drpackiaraj93@gmail.com (P.R.); kabyshev_am_1@enu.kz (A.K.); 5 Centre for Research Impact & Outcome, Chitkara University, Rajpura 140401, Punjab, India

**Keywords:** solid oxide fuel cells (SOFCs), nickel-free anodes, hydrocarbon fuels, ammonia fuels, carbon deposition, exsolution, fuel oxidation

## Abstract

Solid oxide fuel cells (SOFCs) can efficiently convert chemical fuels into electricity, but stable anode operation with hydrocarbon and ammonia fuels remains challenging. Conventional nickel (Ni)-based anodes are prone to carbon deposition, sulfur poisoning, and redox instability, which has stimulated the development of nickel-free (Ni-free) alternatives. This review summarizes the electrochemical conversion pathways of hydrocarbon and ammonia fuels and the limitations of Ni-based anodes under these conditions. Ni-free anode materials are then evaluated against a common set of criteria: surface and defect chemistry, fuel activation, electrochemical performance, carbon tolerance, and structural stability. Within this framework, hydrocarbon activation, carbon formation and removal, and degradation under carbon-based fuels are discussed with particular attention. Among the Ni-free materials, perovskite and double-perovskite oxides provide compositional flexibility with good redox activity, whereas ceria-based fluorite oxides offer favorable oxygen storage and transport properties. Beyond these oxide families, exsolution-derived oxides and oxide composites can generate active interfaces that promote fuel conversion and carbon removal. However, no single material family currently combines high activity, carbon tolerance, and long-term stability under all operating conditions. Therefore, future work should focus on anode operation under realistic fuel conditions, long-term durability and interfacial stability during thermal cycling, and scalable manufacturing routes.

## 1. Introduction

Greenhouse gas (GHG) emissions cause a wide range of environmental impacts by altering weather patterns, contributing to sea-level rise, disrupting ecosystems, accelerating biodiversity loss, and intensifying climate change [1]. These growing environmental concerns have highlighted the urgent need to reduce dependence on fossil fuels and to accelerate the transition toward clean and sustainable energy sources [2]. In this context, fuel cell technology has emerged as a promising alternative for efficient and low-emission energy conversion [3]. A fuel cell is an electrochemical device that converts chemical energy directly into electrical energy [4]. A fuel cell resembles a battery in function, but a battery stores energy internally, whereas a fuel cell requires a continuous fuel supply and cannot store energy itself [5]. Like batteries, fuel cells comprise three main components: an anode, an electrolyte, and a cathode. Fuel cells are classified according to the electrolyte used, and the main types include proton exchange membrane fuel cells (PEMFCs), direct methanol fuel cells (DMFCs), phosphoric acid fuel cells (PAFCs), solid oxide fuel cells (SOFCs), molten carbonate fuel cells (MCFCs), and alkaline fuel cells (AFCs) [6].

**Figure 1:**
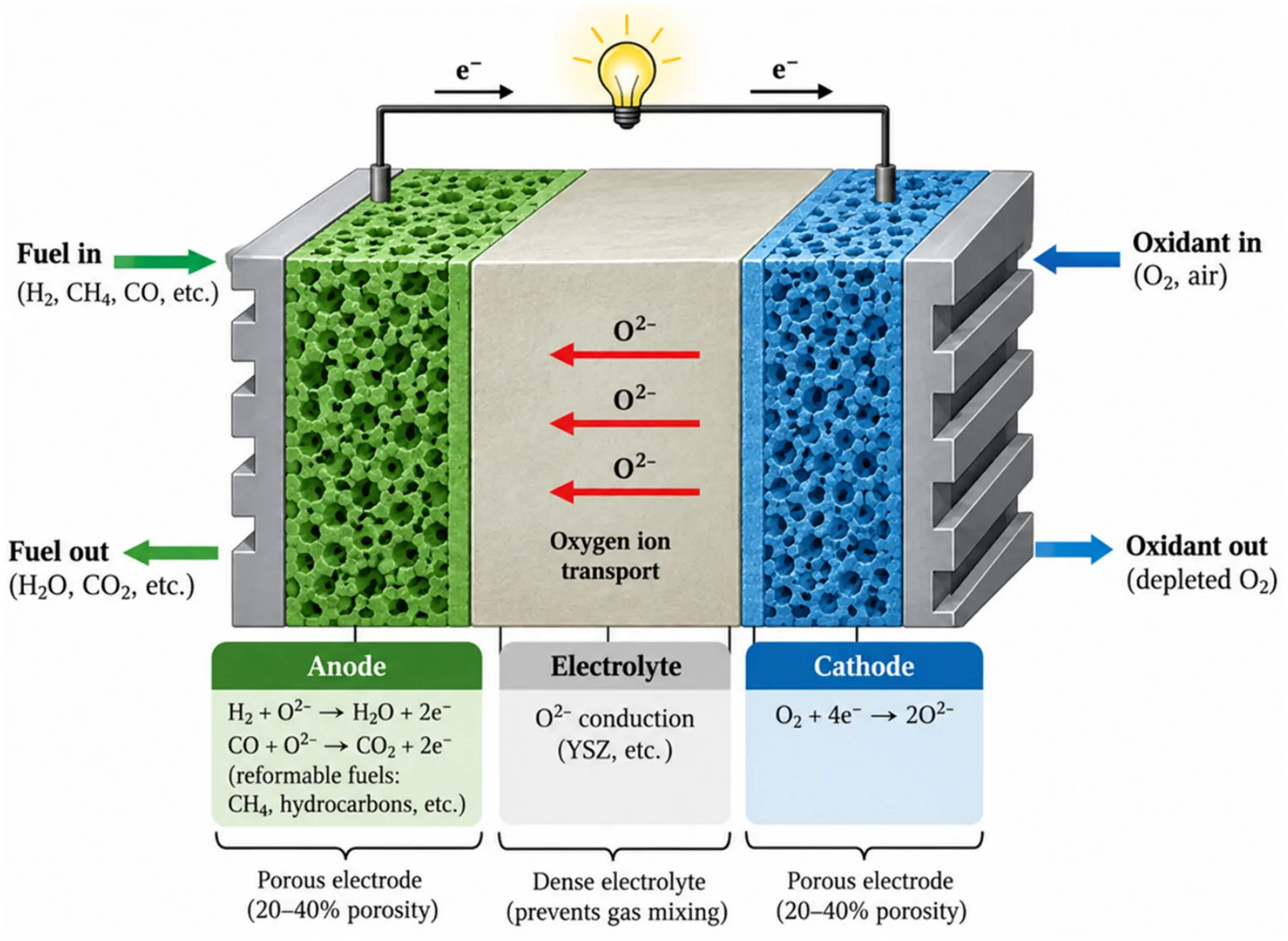
Schematic illustration of the working principle of an oxygen-ion-conducting solid oxide fuel cell (O-SOFC). Figure generated with the assistance of OpenAI ChatGPT (GPT-6).

Among the various fuel cell types, solid oxide fuel cells (SOFCs) offer several advantages, including quiet operation, high efficiency, fuel flexibility (hydrogen, hydrocarbons, and other gases), and relatively low emissions [7]. Their ability to utilize hydrogen, hydrocarbons, biogas, and various industrial waste gases further highlights their potential for flexible and sustainable power generation [8]. SOFCs can be classified into oxygen-ion-conducting SOFCs (O-SOFCs) and proton-conducting SOFCs (H-SOFCs), depending on the type of electrolyte and mobile ionic species. In O-SOFCs, oxygen ions migrate from the cathode to the anode, whereas in H-SOFCs, protons migrate from the anode to the cathode. An SOFC has three main components: the electrolyte, anode, and cathode, each of which must meet specific criteria to achieve high performance. Figure 1 presents a schematic illustration of these components in an O-SOFC. Both electrodes (anode and cathode) require a porosity of 20–40% to allow gas transport, while the electrolyte must remain dense to prevent gas mixing [9]. In addition to total porosity, pore connectivity and permeability are critical elements affecting gas movement through porous materials [10]. The electrodes must also have good electronic and ionic conductivities and remain thermally stable and compatible with the other cell components, while the electrolyte needs good ionic conductivity on its own. SOFCs operate at high temperatures (400–1000 °C) because the solid oxide electrolyte requires thermal activation to achieve sufficient ionic conductivity [11]. This high operating temperature allows SOFCs to use a variety of fuels and to achieve efficiencies above 60%. In this O-SOFC configuration, the hydrogen oxidation reaction (HOR) occurs at the anode, where fuel is supplied, while the oxygen reduction reaction (ORR) occurs at the cathode. The solid electrolyte transports the oxygen ions (O^2−^) generated by the ORR at the cathode toward the anode. At the same time, electrons released at the anode via the HOR travel through an external circuit, generating electrical power before returning to the cathode [12]. This cycle continues as long as fuel and oxidant are supplied. The equations below show the anode and cathode reactions when hydrogen gas is used as the fuel.

(1) $$\mathrm{Anode}:\;H_{2}\;+O^{2-}\text{→ }H_{2}O+2e^{-}$$

(2) $$\mathrm{Cathode}:\;\frac{1}{2}O_{2}+2e^{-}\text{→ }O^{2-}$$

The anode is a key functional component of an SOFC, where fuel oxidation takes place, and the electrons released during the electrochemical reaction are transported through the external circuit to generate electricity. In addition, an SOFC anode must catalyze fuel conversion and electrochemical oxidation and provide sufficient porosity for fuel transport and product removal. For practical operation, the anode must also tolerate a wide range of fuels and operating conditions, including hydrocarbon- and sulfur-containing fuels, and resist carbon deposition and chemical and structural degradation.

Nickel (Ni)-based materials have historically been the preferred anode material because nickel is inexpensive, offers high electronic conductivity, is compatible with common electrolyte materials, and provides adequate mechanical strength. The most widely used anode is the nickel/yttria-stabilized zirconia (Ni-YSZ) cermet, which combines the catalytic activity of nickel toward fuel oxidation with efficient electronic current collection. However, Ni-based anodes have several limitations that become particularly significant when hydrocarbon and other complex fuels are used. In particular, nickel can promote carbon deposition under certain fuel compositions and operating conditions, and sulfur-containing fuels can deactivate the nickel catalyst. Ni-based anodes can also suffer from poor mechanical stability during reduction-oxidation (redox) cycling, an issue that can be more pronounced in anode-supported SOFC designs.

A nickel-based anode can re-oxidize when exposed to oxygen-containing fuels or oxidizing conditions [13]. Because nickel expands during oxidation, repeated redox cycling can generate tensile stress within the anode, which may lead to anode cracking and damage to the adjacent thin electrolyte [14]. Such degradation can shorten cell lifetime and reduce electrochemical efficiency [15]. These limitations have motivated the development of alternative anode materials that offer improved fuel compatibility and structural stability. Tao and Irvine reviewed alternative oxide anodes, emphasizing their structural properties, electrical conductivity, redox stability, and compatibility with electrolytes [16]. Ge et al. examined anode materials for direct hydrocarbon utilization, highlighting the importance of carbon resistance and fuel flexibility [17]. More recently, Xia et al. discussed advances in perovskite ceramic anodes, particularly compositional modification strategies aimed at improving catalytic performance and resistance to carbon deposition and sulfur poisoning [18].

**Figure 2:**
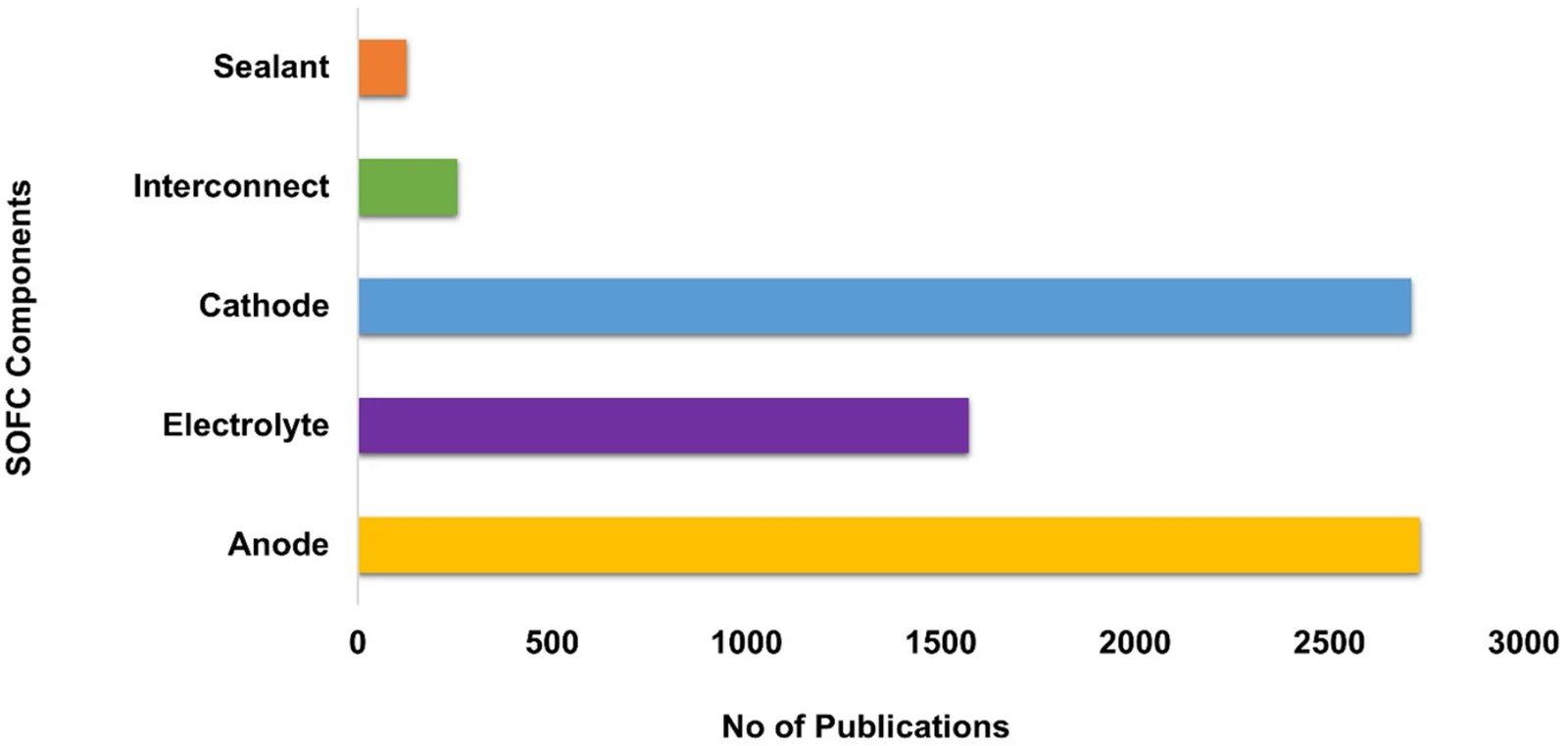
Number of publications associated with different SOFC components according to Scopus data.

According to Scopus data, 2733 documents were retrieved on SOFC anodes. Figure 2 compares the number of publications associated with the major SOFC components. The anode has the highest publication count in the dataset, followed closely by the cathode, while the electrolyte has a substantially lower number of publications. The interconnect and sealant show considerably fewer publications. Although publication numbers depend on the database and search strategy used, the distribution shown in Figure 2 indicates sustained research interest in SOFC anodes and the continued development of anode materials. The performance of alternative anodes depends strongly on material composition, microstructure, surface chemistry, fuel conversion, and stability under operating conditions, which motivates a systematic comparison of these materials.

Although several reviews have addressed alternative SOFC anodes, a comprehensive assessment is still needed to understand how material characteristics influence electrochemical performance and degradation under different fuel conditions. Existing reviews provide valuable insights into individual aspects of anode development, but the relationships among surface and defect chemistry, oxygen transport, fuel activation, carbon formation and removal, and structural stability during operation remain insufficiently discussed and compared across Ni-free anode families. To address this gap, this review provides an integrated comparison of Ni-free anode materials for hydrocarbon- and ammonia-fueled SOFCs by examining their material properties, fuel-dependent electrochemical behavior, and degradation mechanisms, with particular attention to carbon formation and removal during operation on carbon-based fuels. In addition, a material selection framework is proposed that integrates catalytic and chemical properties, structural and redox stability, and electrical, ionic, and mechanical characteristics. This framework supports systematic assessment of the suitability of Ni-free anode materials in terms of performance, durability, and compatibility with specific fuels and operating conditions. The remainder of this review is structured as follows. Section 2 describes the review methodology, and Section 3 discusses hydrocarbon- and ammonia-based fuels and their reaction pathways. Section 4 establishes the criteria for selecting anode materials, and Section 5 summarizes conventional Ni-based anodes as a reference and evaluates the main classes of Ni-free anode materials. Section 6 examines how Ni-free anodes interact with carbon-based fuels, focusing on hydrocarbon activation, carbon formation and removal, and degradation mechanisms. Section 7 outlines outstanding challenges and future research directions, and Section 8 presents the main conclusions.

## 2. Review Methodology

The literature discussed here was drawn mainly from peer-reviewed journal articles and reviews indexed in Scopus and published between 2020 and 2026, using search terms covering SOFC anodes, hydrocarbon and ammonia fuels, nickel-free anode chemistries, ceria-based composites, perovskite oxides, and spinel oxides. Priority was given to studies reporting cell- or half-cell-level electrochemical performance, carbon deposition or sulfur-tolerance behavior, or redox stability under hydrocarbon- or ammonia-relevant conditions; earlier foundational or highly cited work was included where it provided necessary context.

## 3. Hydrocarbon- and Ammonia-Based Fuels for SOFCs

Hydrogen is an ideal fuel for energy conversion, but it is not naturally available, and its production and storage pose significant challenges. One advantage of the SOFC is the range of fuels it can use directly, without the need for an external reformer or fuel processor [19]. Fuels such as natural gas, biogas, methane, ethanol, and ammonia can be used at the high operating temperatures typical of SOFCs. SOFCs fueled by hydrocarbons or ammonia are expected to reach wider commercial applications because these fuels are inexpensive, easy to store, and have narrower explosive limits than hydrogen. The following subsections discuss methane, ammonia, and alcohol-based fuels as representative SOFC fuels.

### 3.1. Methane

Among hydrocarbon fuels, methane is particularly attractive. It is abundant in natural gas and methane hydrates and can also be produced from biomass or carbon dioxide (CO_2_) through various conversion routes. Several chemical and electrochemical reactions occur during methane utilization in SOFCs and are typically grouped as (i) reforming reactions, including steam reforming and dry reforming; (ii) the water–gas shift reaction (WGS); (iii) partial oxidation (POX); (iv) direct methane oxidation; (v) hydrocarbon oxidation; (vi) coking, or carbon deposition; and (vii) complete combustion. Table 1 lists the main methane reactions occurring at the SOFC anode. Reforming reactions are especially important because they convert methane into hydrogen-rich gas mixtures. For example, in steam reforming, the CO produced can be further converted to CO_2_ through the water–gas shift reaction, which increases the H_2_ yield.

**Figure 3:**
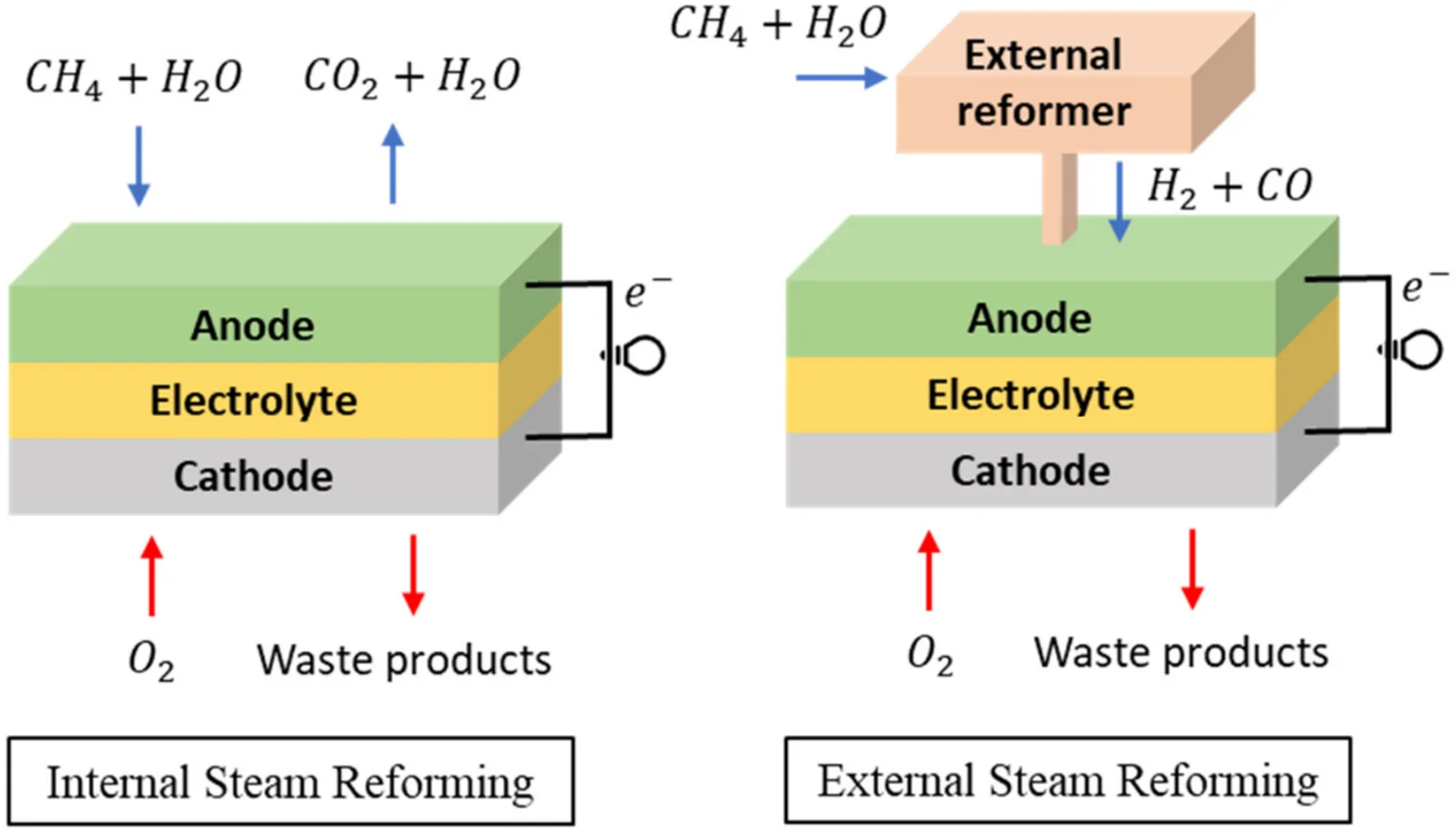
Internal and external steam reforming reaction process.

Steam reforming (SR) is a process in which a hydrocarbon reacts with steam (H_2_O) at high temperature to produce hydrogen (H_2_) and carbon monoxide (CO). The resulting H_2_ and CO are fed directly to the anode, where they are electrochemically oxidized to generate electricity. Reforming can be performed either internally or externally. Reforming the fuel internally (internal SR) eliminates the need for an external reformer and reduces the external energy input required for reforming [19]. However, internal SR cools the stack unevenly because reforming proceeds rapidly near the fuel inlet, producing steep local temperature gradients that can damage the anode’s mechanical integrity. Therefore, matching the reforming rate to the electrochemical reaction rate is essential to limit this effect [19]. In contrast, external SR converts the fuel into hydrogen-rich gas in a separate reformer before it reaches the anode. Since reforming occurs externally, the cell design is simpler, which can improve durability and lifespan. At the same time, the implementation of an additional reformer increases overall cost. Figure 3 illustrates the difference between internal and external SR.

Another reforming route is dry reforming, in which CO_2_ replaces steam as the reforming agent. Because it consumes both CH_4_ and CO_2_, this reaction can contribute to mitigating greenhouse gas emissions. It is strongly endothermic and requires high temperatures (750–1000 °C) and low pressure [20]. Syngas can also be produced by partial oxidation (POX), in which a limited amount of oxygen reacts with the hydrocarbon fuel to yield a mixture of H_2_, CO, and CO_2_. The process is termed ‘partial’ because only a fraction of the fuel is oxidized, and the resulting syngas is supplied to the anode for power generation. POX does not require steam, which simplifies the system. The reaction is also exothermic, and the heat released helps maintain the SOFC operating temperature [21].

The water–gas shift (WGS) reaction occurs alongside steam reforming and further increases the hydrogen content of the fuel by converting CO and H_2_O into CO_2_ and H_2_. This improves the fuel composition and reduces the CO content [22]. Electrochemical oxidation of hydrogen occurs at the anode, where hydrogen reacts with oxygen ions to form water and release electrons, which generate an electric current. This is the principal SOFC reaction and occurs at the triple-phase boundary (TPB), where the gas phase, electrode, and electrolyte meet, through several steps: hydrogen diffusion, adsorption on the electrode surface, charge transfer, and water formation [23]. At the cathode, the ORR reduces molecular oxygen (O_2_) from air to oxygen ions (O^2−^), a process that proceeds through oxygen adsorption, reduction, and ion transport and is equally essential to cell operation. Direct methane (DM) oxidation occurs when methane is used directly as the fuel. At the anode and at high temperatures, methane is either oxidized electrochemically to CO_2_ and H_2_O or partially reformed internally to H_2_ and CO, which are subsequently oxidized. One drawback of this pathway is carbon deposition on the anode surface, which can degrade cell performance. Methane decomposition refers to the breakdown of CH_4_ into carbon and H_2_. The hydrogen released from this process participates in the electrochemical reactions of the SOFC, but the carbon formed can accumulate as deposits and lead to coking [24].

**Figure 4:**
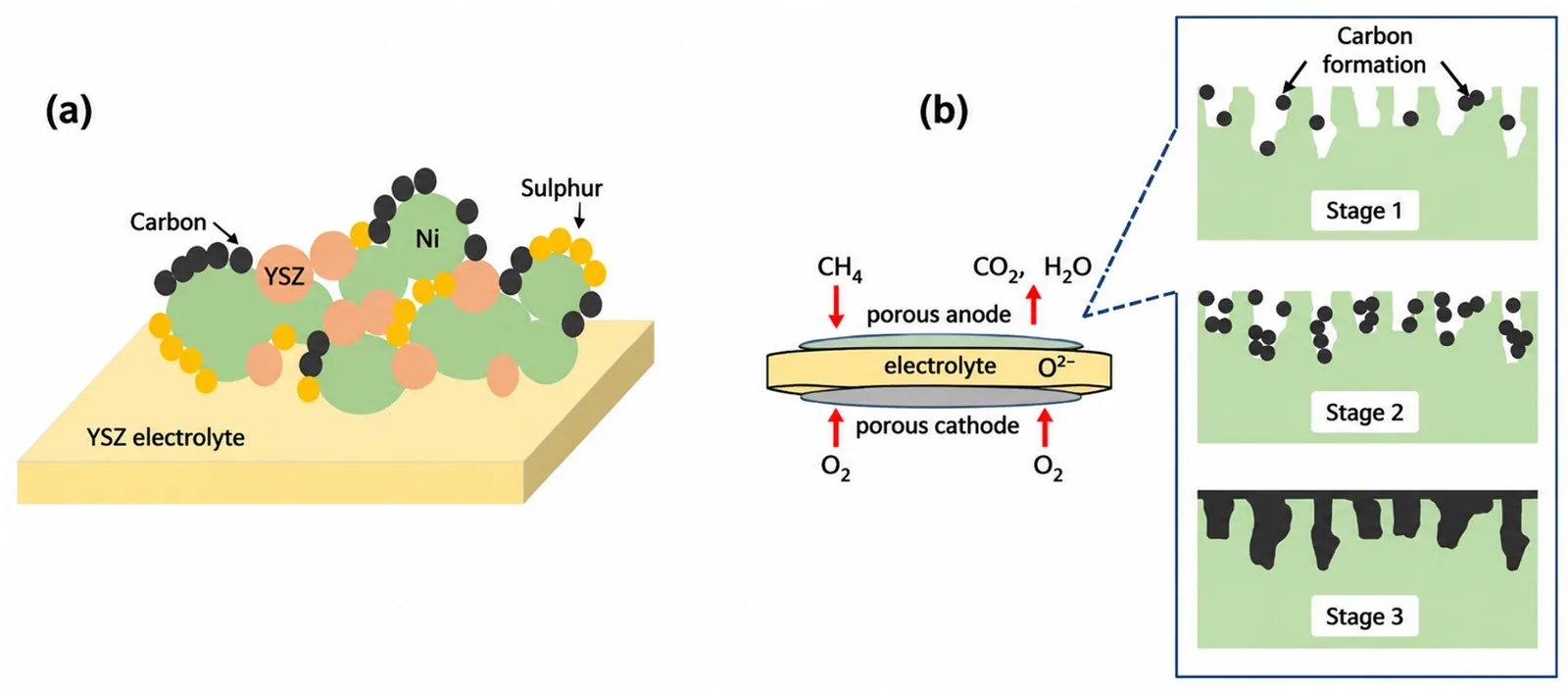
(**a**) Carbon deposition and sulfur poisoning; (**b**) three stages of carbon formation.

Most reforming and electrochemical oxidation reactions occur at the anode, where the fuel is supplied and processed. Methane can also be oxidized electrochemically at the anode by O^2−^ ions transported from the cathode through the electrolyte, which bypasses the reforming step and converts methane directly into useful products and electrons [25]. The use of hydrocarbon fuels in SOFCs nonetheless presents two major challenges: carbon deposition on the anode and sulfur poisoning. Carbon deposition arises mainly from methane cracking, which is also known as methane decomposition. The solid carbon produced can deposit on the anode and degrade SOFC performance by blocking the TPB [26]. Most hydrocarbon fuels also contain sulfur compounds, which poison the anode catalyst by blocking active sites and reducing catalytic and electrochemical activity. Figure 4a illustrates carbon deposition and sulfur poisoning on the anode, while Figure 4b shows the three stages by which carbon builds up on the anode surface. In stage 1, small amounts of carbon begin to deposit at the active catalytic sites without covering the full anode surface. During this stage, current density can rise slightly because the deposited carbon adds some conductivity. In stage 2, carbon at these sites starts to interconnect into layers that eventually cover the entire anode surface. Fuel gases can still diffuse through the porous carbon layer, but the growing coverage progressively reduces the active reaction area at the TPB and slows the reaction rate.

In stage 3, the deposited carbon forms a thick layer that eventually blocks fuel gas from reaching the TPB, sharply reducing the reaction rate. As a result, SOFC output power can progressively decline with time [27]. In addition to carbon deposition, microstructural evolution can degrade Ni-based anodes. For example, nickel/gadolinium-doped ceria (Ni-GDC) anodes showed declining electrochemical performance within the first 10 h of operation, which was attributed to rapid microstructural change [28]. During operation, GDC migrated throughout the anode and formed a thin layer over the Ni particles, and this layer was thinner near the electrolyte and thicker near the current collector. In that study, 3D reconstructions showed that most Ni grains were covered by this GDC layer after 100 h of operation. Ni coarsening (grain growth) was especially pronounced near the electrolyte, which contributed to performance loss through reduced TPB density and increased polarization resistance in that region. Together, these degradation pathways limit the long-term operation of Ni-based anodes on hydrocarbon fuels and motivate the development of Ni-free alternatives.

### 3.2. Ammonia

Ammonia (NH_3_) is drawing growing interest as a hydrogen carrier and SOFC fuel, owing to its high hydrogen content (17.6 wt%) and ease of storage and transport [29]. Its advantages include:

- No CO_2_ emissions at the point of use
- Easier storage as a liquid than hydrogen or methane, owing to its higher boiling point (–33 °C, compared with –253 °C for hydrogen and –160 °C for methane)
- Large-scale production, as one of the most widely produced chemicals worldwide (up to ~180 million tons annually)
- High volumetric energy density (11.38 GJ m^–3^)
- Low flammability
- A relatively narrow flammability range (16–25% in air)

However, ammonia also presents safety risks, including:

- Toxicity upon inhalation
- Possible fatality within 30 min at 2000–3000 ppm in air
- Severe health effects at concentrations above 5000 ppm

Nevertheless, these risks are limited and manageable, and should not stand in the way of developing ammonia as an energy source. When ammonia decomposes, it yields hydrogen (H_2_) and nitrogen (N_2_) according to the following equation:

(3) $${2\mathrm{NH}}_{3}\text{ → }{3H}_{2}\;+\;N_{2}$$

The hydrogen produced is used in electrochemical reactions to generate electricity, while the nitrogen is released as an inert byproduct. In an oxygen-ion-conducting SOFC, ammonia can undergo electrochemical oxidation at the anode, as represented by Equations (4) and (5). In addition, a secondary chemical reaction involving ammonia and nitric oxide may occur, as shown in Equation (6). Oxygen reduction occurs at the cathode, as represented by Equation (7) [30]:

(4) $${2\mathrm{NH}}_{3}\;+\;{3O}^{2-}\text{ → }N_{2}\;+\;{3H}_{2}O\;+\;6e^{-}\;(\mathrm{anodic}\;\mathrm{reaction})$$

(5) $${2\mathrm{NH}}_{3}\;+{5O}^{2-}\text{ →}\;2\mathrm{NO}\;+{3H}_{2}O+10e^{-}\;(\mathrm{anodic}\;\mathrm{reaction})$$

(6) $${2\mathrm{NH}}_{3}\;+3\mathrm{NO}\text{ → }\frac{5}{2}N_{2}+{3H}_{2}O\;(\mathrm{secondary}\;\mathrm{chemical}\;\mathrm{reaction})$$

(7) $$O_{2}\;+{4e}^{-}\text{ → }{2O}^{2-}\;(\mathrm{cathodic}\;\mathrm{reaction})$$

The overall reaction in a direct ammonia-fueled SOFC is given by Equation (8):

(8) $${2\mathrm{NH}}_{3}\;+\;\frac{3}{2}O_{2}\text{ → }N_{2}\;+\;{3H}_{2}O\;$$

One challenge in using ammonia in SOFCs is anode degradation when nickel (Ni) serves as the anode material. This degradation stems from nitridation of the nickel catalyst, which drives microstructural change in the anode [29]. Nitridation occurs when ammonia reacts with the nickel catalyst to form nickel nitride (Ni_3_N), as shown in the following equation:

(9) $$6\mathrm{Ni}\;+\;{2\mathrm{NH}}_{3}\text{ → }{2\mathrm{Ni}}_{3}N\;+\;{3H}_{2}$$

The resulting Ni_3_N is not fully stable and can break down again under a reducing environment or at high temperature. As nitriding proceeds, Ni_3_N forms but can also decompose back into metallic nickel during ammonia decomposition. Because Ni_3_N occupies a larger volume than metallic nickel, this cycle causes local expansion within the anode. Once Ni_3_N decomposes, the resulting volume change leaves pits on the nickel surface. Repeated cycles of nitriding and reduction therefore lead to irreversible changes in the anode microstructure, which in turn reduces cell performance [31]. Wang et al. lowered the operating temperature from 800 °C to 700 °C and found that nitridation at this lower temperature increased the anode thickness [32]. Nitridation caused volume expansion, reduced electrode porosity, and enlarged the contact area, which in turn intensified further nitridation. The greater thickness also lowered the average power density and the fuel-to-energy conversion efficiency. Taken together, these thickness- and temperature-related changes adversely affected SOFC performance, stability, and efficiency [31]. Sorcar et al. impregnated iron (Fe) into a Ni-anode to lower the cell’s internal resistance and reduce anodic overpotential [33]. The added Fe also enhanced hydrogen oxidation. In this work, a new anode for direct anion-exchange SOFCs was developed by supporting Ni and Fe nanoparticles on a lanthanum titanium oxynitride support, combined with copper- and gadolinium-doped ceria (GDC). The resulting cell reached a current density of 939 mA cm^−2^ and power density of 228 mW cm^−2^ at 750 °C. The authors reported that this oxynitride anode performed well with no sign of physical damage or delamination, in contrast to conventional YSZ-based anodes.

### 3.3. Alcohol-Based Fuels

Alcohols (ethanol and methanol) are attractive liquid fuels for SOFCs because they are easier to store and transport than hydrogen and can be reformed either internally or in an upstream reformer to produce a hydrogen-rich gas [34]. Among these fuels, ethanol is particularly relevant because it can be produced from biomass, although it contains both C–C and C–O bonds, which makes its reforming chemistry more complex than that of methanol [35]. Internal reforming can reduce the need for a separate external reformer and associated balance-of-plant components, potentially reducing system size and complexity [36]. However, this advantage should not be interpreted as evidence that alcohol-fueled SOFCs are inherently simpler or more durable. The practical feasibility of internal alcohol reforming is strongly constrained by catalyst deactivation, carbon formation, reforming kinetics, and the substantial heat requirement of steam reforming [37].

For ethanol, the ideal overall steam reforming reaction can be expressed as

C_2_H_5_OH + 3H_2_O ⇌ 2CO_2_ + 6H_2_     ΔH_298_ = +173.5 kJ mol^−1^ (10)

Thus, ethanol steam reforming (ESR) is strongly endothermic and requires continuous heat input to sustain conversion [38]. The reaction can also proceed through partial reforming and several parallel reactions, including ethanol dehydrogenation, dehydration, decomposition, methanation, and the water–gas shift reaction [39]. For example,

C_2_H_5_OH + H_2_O ⇌ 2CO + 4H_2_     ΔH_298_ = +256 kJ mol^−1^ (11)

and

C_2_H_5_OH ⇌ C_2_H_4_ + H_2_O     ΔH_298_ = +45 kJ mol^−1^ (12)

The latter reaction is particularly important because ethylene formed during ethanol conversion can undergo further reactions leading to carbonaceous deposits. In practice, the experimentally observed product distribution is generally more complex than the ideal reaction suggests. Feng et al. outlined the various reaction pathways in ethanol steam reforming, highlighting the production of intermediates or by-products such as CO, CH_4_, acetaldehyde, and ethylene [40]. Ogo and Sekine highlighted the impact of catalyst composition and reaction mechanisms on selectivity of products and coke formation [41]. Simultaneously, Sharma et al. examined strategies for catalyst modification to reduce carbonaceous buildup and enhance catalyst stability during ethanol steam reforming [42].

The endothermicity of ESR presents an important limitation for direct internal reforming in SOFCs [43]. Although the high operating temperature of an SOFC provides a potentially convenient heat source, the reforming reaction consumes heat locally and can disturb the thermal balance of the electrochemical process. This can produce substantial temperature gradients within the anode and reforming region, particularly when ethanol conversion occurs rapidly near the fuel inlet. Therefore, eliminating an external reformer does not eliminate the energy requirement associated with fuel processing. Instead, the heat demand is transferred into the SOFC stack and must be supplied through internal heat generation, heat recovery, or thermal coupling with the electrochemical reactions. Thermodynamic and energy–exergy analyses have identified heat management as a major consideration in integrating ethanol reforming with SOFC operation [44].

This heat requirement also creates a trade-off between reforming performance and thermal management. Increasing the reforming temperature generally promotes ethanol conversion and suppresses some incompletely reformed products, but higher temperature can simultaneously accelerate catalyst sintering and other degradation processes [45]. Recent reviews of ESR catalysts report that high-temperature operation is associated with catalyst deactivation through both carbon deposition and sintering, while insufficient temperature can lead to incomplete conversion and increased formation of undesired intermediates [40]. Therefore, a suitable operating temperature cannot be selected solely on the basis of maximum ethanol conversion, but must also account for heat demand, carbon tolerance, catalyst stability, and the thermal limits of the SOFC components.

Carbon formation is an additional limitation that is particularly significant for Ni-containing anodes [27]. Ethanol can undergo dehydration to form ethylene, which may subsequently participate in secondary reactions such as dimerization [46]. Carbon deposition can also occur during ethanol dehydration, and its extent is influenced by reaction conditions and surface reaction pathways [47]. Other carbon-forming routes may involve CO disproportionation or decomposition of hydrocarbon intermediates generated during incomplete ethanol reforming. The resulting deposits can cover catalytically active sites, block pores, restrict fuel transport, and progressively reduce the electrochemically active area. Márquez et al. investigated the deactivation of a Ni/La_2_O_3_–Al_2_O_3_ catalyst at 500–650 °C using steam-to-ethanol ratios of 3–9 and found that catalyst deactivation was primarily associated with coke deposition [48]. In particular, encapsulating coke progressively covered the active Ni sites, while ethanol, acetaldehyde, and ethylene were identified as important precursors of the deposited carbon. The extent and nature of coke formation also depended on the operating conditions, indicating that catalyst stability cannot be assessed independently of temperature and steam-to-ethanol ratio. Similarly, Alberton et al. examined carbon formation during ESR over Ni/Al_2_O_3_ catalysts [49]. Their results showed that coke could form through several pathways, including the Boudouard reaction, methane decomposition, and polymerization of ethylene produced through ethanol dehydration. The study distinguished between filamentous carbon and encapsulating carbon: filamentous carbon did not necessarily cause immediate loss of catalytic activity, whereas encapsulating carbon covered the active Ni phase and promoted catalyst deactivation. The study also demonstrated that increasing the temperature and steam-to-ethanol ratio reduced carbon deposition, highlighting the importance of operating conditions in controlling catalyst stability.

The severity of this problem becomes more evident under direct ethanol feeding to conventional Ni-based SOFC anodes. Laosiripojana et al. reported that ethanol could not be directly supplied to a Ni/YSZ anode without substantial carbon formation, even at high steam content and elevated operating temperature [50]. Incomplete ethanol reforming generated ethane and ethylene, which were identified as important contributors to the high carbon-formation rate [50]. In contrast, pre-reforming ethanol over a separate Ni/CeO_2_–ZrO_2_ catalyst before feeding the resulting gas to the Ni/YSZ anode reduced carbon deposition compared with direct feeding [51]. This result suggests that the compatibility of ethanol with SOFC operation depends not only on the thermodynamic possibility of reforming but also on where and how the reforming reactions occur. Experimental studies have nevertheless demonstrated that internal reforming can be achieved when the anode is provided with an appropriate catalytic function. Dogdibegovic et al. reported peak power densities of approximately 1.0 W cm^−2^ at 650 °C using an ethanol–water blend and 1.4 W cm^−2^ at 700 °C with anhydrous ethanol in a metal-supported SOFC containing a Ni–samarium-doped ceria (SDC) reforming catalyst [34]. Initial tests with the ethanol–water mixture showed stable operation for 100 h at 700 °C and 0.7 V without detectable carbon deposition in the Ni–SDC anode. However, these results should be interpreted as evidence of the effectiveness of a specific anode/catalyst configuration rather than proof that ethanol is intrinsically carbon-tolerant. The observed stability depended on catalyst architecture and operating conditions, including the presence of water.

The importance of catalyst configuration is further demonstrated by Steil et al., who reported more than 700 h of stable operation on dry ethanol when a highly active Ir–gadolinia-doped ceria (Ir-CGO) catalytic layer was incorporated over a conventional Ni-based anode [52]. In the absence of this catalytic layer, the cell experienced rapid degradation associated with carbon deposition, whereas the ceria-based layer promoted ethanol conversion using electrochemically generated steam and suppressed carbon accumulation. Similarly, more recent work using an integrated Ru–GDC internal reforming layer demonstrated stable ethanol-fueled SOFC operation while separating much of the ethanol reforming function from the electrochemical reaction sites [53]. However, increasing ethanol concentration was found to reduce ethanol conversion and potentially increase the risk of carbon deposition, illustrating the sensitivity of internal reforming to fuel concentration and operating conditions.

Water addition can mitigate these limitations by increasing the steam-to-carbon ratio and promoting carbon removal, but it introduces another system-level trade-off. Higher steam content generally suppresses carbon formation and favors ethanol conversion, yet excessive steam dilutes the fuel, lowers the effective fuel concentration, and increases the energy required for steam generation and vaporization. In an experimental study, Hassanzadeh et al. demonstrated that the operating conditions strongly influenced ethanol conversion and catalyst stability during combined steam and CO_2_ reforming of ethanol [45]. Their response-surface analysis identified temperature as the dominant parameter, while the CO_2_/H_2_O ratio controlled the H_2_/CO ratio. Under optimized conditions at 900 °C, the Ni–Cu/ZrO_2_ catalyst achieved more than 90% ethanol conversion and maintained stable activity for approximately 950 min, with improved resistance to sintering and carbon deposition compared with Ni/ZrO_2_. These results highlight the importance of optimizing steam-containing reforming conditions to balance conversion, carbon tolerance, and catalyst stability. Thermodynamic analysis of ethanol/steam mixtures has indicated that a steam-to-ethanol ratio of approximately 3–6 can provide a compromise between carbon suppression and excessive steam-related losses under certain conditions [54]. Thus, increasing the water content cannot be regarded as a universal solution to carbon deposition.

Catalyst deactivation during ESR is therefore governed by multiple interacting mechanisms rather than carbon deposition alone. Carbon accumulation can block active sites, while high-temperature operation can promote metal sintering and loss of active surface area [55]. In addition, incomplete reforming can produce CH_4_ and C_2_ hydrocarbons that alter the downstream fuel composition and can introduce additional carbon-forming pathways. Experimental studies show that catalyst stability during ethanol steam reforming is strongly influenced by metal particle size, metal–support interactions, and support properties. For example, Sánchez-Greluk et al. found that the dispersion and crystallite size of Ni and Co on CeO_2_ affected catalyst properties and deactivation, while the oxidation state of the CeO_2_ support was also influenced by metal particle size [56]. In another experimental study, Meng et al. demonstrated that strong Rh–Ni/TiO_2_ metal–support interactions, together with oxygen-vacancy-rich interfaces, enhanced ESR activity and maintained stable operation for 300 h [57]. These results indicate that active-metal dispersion, metal–support interactions, and support oxygen chemistry are closely coupled to ESR catalyst stability. These factors are particularly relevant to SOFC anodes because catalyst deactivation can simultaneously reduce reforming activity and alter the composition of the gas reaching the electrochemically active region.

The practical implication is that internal reforming of alcohol fuels should be regarded as a coupled fuel-processing and electrochemical process, rather than simply as a method for eliminating an external reformer. A successful design must simultaneously control ethanol conversion, carbon formation, catalyst stability, heat transfer, steam utilization, and the composition of the reformate supplied to the electrochemical reaction zone. One strategy is to introduce a dedicated catalytic layer upstream of or within the anode to promote reforming while spatially separating the most carbon-sensitive electrochemical regions from the primary ethanol conversion zone. Recent NiMo/Ce–ZrO_2_ and Ru–GDC internal-reforming configurations demonstrate this approach by increasing reforming activity and improving resistance to coking under direct ethanol feed conditions [58].

Overall, alcohol-based fuels offer the practical advantages of liquid-fuel handling and the possibility of internal reforming, but these advantages are accompanied by substantial chemical and thermal penalties. Ethanol is more difficult to reform cleanly than a simple one-carbon alcohol because of its multiple reaction pathways and the formation of carbon-forming intermediates such as ethylene. The strongly endothermic nature of ESR also imposes a significant heat-management requirement, while high-temperature operation can accelerate catalyst sintering and deactivation. Consequently, the principal challenge is not simply to achieve high ethanol conversion or high initial SOFC power density, but to maintain stable reforming and electrochemical performance over extended operation without carbon accumulation or excessive thermal gradients. This makes catalyst architecture, steam-to-ethanol ratio, heat integration, and carbon-removal capability central considerations in the development of alcohol-fueled SOFC anodes.

## 4. Framework for Anode Material Selection

**Figure 5:**
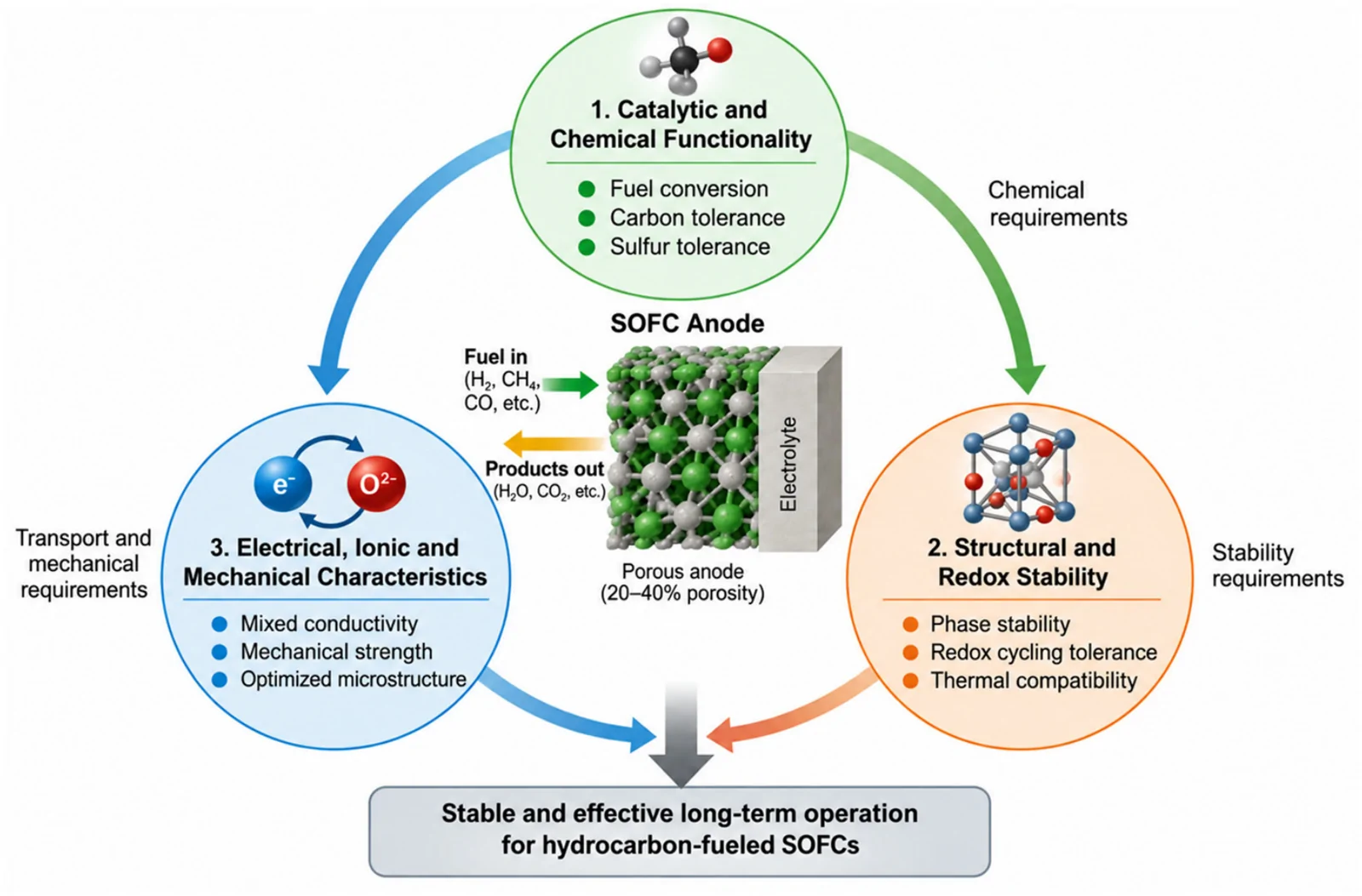
Framework for anode material selection based on catalytic and chemical functionality, structural and redox stability, and electrical, ionic, and mechanical characteristics. Figure generated with the assistance of OpenAI ChatGPT (GPT-6).

The anode is a key component in SOFC operation, providing electrochemical oxidation, current collection, and structural support. The selection of a suitable anode material for direct hydrocarbon operation must consider the physicochemical, electrochemical, and thermomechanical aspects together to ensure stable long-term operation. Figure 5 summarizes this framework and depicts the relationship between the three groups of criteria used for selecting anode materials. Instead of assessing individual material properties separately, this framework offers a unified method for comparing Ni-free anode candidates by considering their catalytic performance, fuel compatibility, redox stability, and transport characteristics. It also emphasizes the trade-offs among these characteristics, enabling researchers to identify suitable materials based on particular fuel compositions and operational conditions. For example, an anode featuring strong catalytic activity may not be the optimal choice if it shows poor carbon resistance or structural instability during extended operation.

### 4.1. Catalytic and Chemical Functionality

An anode’s catalytic ability determines how effectively it converts fuel and, in turn, contributes to overall cell performance. An effective anode needs sufficient catalytic activity for hydrogen and hydrocarbon oxidation while also promoting reforming and partial oxidation. In hydrocarbon-fueled SOFCs, the anode must promote fuel conversion while suppressing excessive methane cracking and carbon-forming side reactions, such as the Boudouard reaction (2CO → C + CO_2_) [59]. Chemical functionality is closely tied to carbon tolerance [60]. Materials that supply lattice oxygen, such as doped ceria or certain perovskites, can oxidize carbon species in situ and reduce coking, while adding oxygen-storage or oxygen-diffusive phases can strengthen this self-cleaning effect. Sulfur tolerance is also important because trace sulfur compounds in real fuels can damage catalytic sites. Nickel-based anodes are highly vulnerable to sulfur poisoning, whereas alternative materials such as Cu-, Fe-, and Mo-based perovskites and spinel oxides bind sulfur more weakly and can maintain catalytic activity [61]. Overall, an anode’s chemical properties need to support effective hydrocarbon oxidation, resistance to carbon buildup, and tolerance to sulfur-containing compounds while preserving catalytic integrity during extended operation.

### 4.2. Structural and Redox Stability

Long-term stability under redox and thermal cycling strongly influences cell lifespan. During transient operation or fuel disruption, the anode can undergo cyclic oxidation and reduction, leading to microstructural damage and mechanical breakdown [62]. In Ni-based systems, the sizable volume change between Ni and NiO during redox cycling causes mechanical stress, grain coarsening, and delamination. Oxide-based anodes such as doped ceria, perovskites, and spinel structures can offer greater structural durability, owing to their stable crystal lattices and, in some systems, their capacity for metal exsolution under reducing conditions [63]. Thermal and chemical compatibility with the electrolyte and interlayer materials is also essential for preserving interfacial integrity. In particular, the anode’s thermal expansion coefficient (CTE) should remain close to that of the electrolyte to minimize cracking or distortion during temperature changes [64]. In summary, redox and thermal stability help to ensure that the anode preserves its phase composition, microstructure, and adhesion during prolonged operation, allowing for dependable long-term performance.

### 4.3. Electrical, Ionic and Mechanical Characteristics

Efficient anode operation depends on the simultaneous transport of electrons and oxygen ions within the active region. Mixed ionic-electronic conductors (MIECs) can extend the electrochemically active area beyond the usual triple-phase boundary (TPB), which can improve reaction kinetics and fuel utilization [65]. For instance, perovskites and doped ceria anodes provide both electronic and ionic conduction pathways, whereas conventional Ni-YSZ systems rely strongly on TPB-associated reaction sites. Mechanical strength and microstructural design are also important because they govern gas diffusion, current collection, and the cell’s overall mechanical integrity. An optimized anode microstructure typically maintains 20–40% interconnected porosity, balancing fuel accessibility against stability under thermal stress. Sintering conditions, grain interconnectivity, and pore structure all require careful control to balance mechanical strength against efficient mass transport [66].

## 5. Anode Material Classes for Hydrocarbon- and Ammonia-Fueled SOFCs

### 5.1. Nickel-Based Anodes (Baseline)

Nickel (Ni) meets most of the requirements for an anode material, owing to its low cost, high electrochemical activity, good electrical conductivity, thermal and chemical stability, compatibility with various fuels, and strong mechanical properties [67]. However, nickel also presents several problems: carbon deposition is a major concern when Ni-based anodes are operated with hydrocarbon-containing fuels such as biogas [68]. Other limitations include sulfur poisoning, agglomeration of nickel particles at high temperatures, and thermal expansion mismatch with the electrolyte, which can generate mechanical stresses during thermal cycling [67]. Substantial improvements have addressed these problems over the years. For example, combining nickel with yttria-stabilized zirconia (YSZ) increases the number of reaction sites and improves electrode performance. Porous Ni-YSZ cermets are typically produced by reducing a sintered NiO-YSZ composite together with the other cell components [69]. The resulting cermet remains the most widely used anode for high-temperature SOFCs because it satisfies most ideal-anode criteria at once. The nickel in the cermet acts as an excellent electrocatalyst with high electronic conductivity, while the surrounding YSZ inhibits nickel coarsening during operation [68]. The CTE of Ni-YSZ is also close to that of the YSZ electrolyte and can be fine-tuned further by adjusting its composition. Since its introduction, this material has seen steady advances in composition, design, and fabrication.

To prevent coking, one study developed a simple, efficient phase inversion-impregnation method to create BaO-decorated Ni-YSZ (8 mol% YSZ) anode-supported tubular SOFCs [70]. BaO nanoislands smaller than 100 nm were distributed uniformly within the Ni-YSZ anode, and the cell reached peak power densities of 0.30 W/cm^2^ and 0.22 W/cm^2^ at 800 °C in wet hydrogen and methane fuel, respectively [70]. The authors reported that the BaO-decorated anode achieved markedly better long-term stability in methane than the non-decorated version. Density functional theory (DFT) calculations further indicated that the BaO nanoislands adsorbed and dissociated H_2_O molecules, generating OH species that help remove carbon deposits. They concluded that this phase inversion-impregnation route reduces both time and cost relative to traditional methods while offering a new path to anti-coking SOFC anodes. In a related approach, a dual-layer Ni-YSZ/Ni-Fe anode for SOFCs was synthesized by phase-inversion tape casting, reaching a maximum power density of 1070 mW/cm^2^ at 800 °C, higher than a single-layer anode made by the traditional hot isostatic pressing method [71]. The improvement was attributed to straighter pore paths in the anode, which improve gas transport, reduce concentration polarization, and boost the efficiency of electrochemical reactions. Redox stability testing also showed that the dual-layer cell remained stable over eight redox cycles, with power density decreasing only slightly, from 690 mW/cm^2^ to 636 mW/cm^2^. In contrast, the single-layer anode made by the traditional method failed after just one cycle because of cracking.

Researchers have also explored alternatives such as Ni-gadolinium-doped ceria (Ni-GDC) in the search for materials that operate at lower temperatures while offering better electrochemical performance and durability. Ni-GDC is known for operating at relatively low temperatures (around 600–800 °C), which makes it attractive for extending cell life and limiting degradation [72]. Padinjarethil et al. compared Ni-YSZ and Ni-GDC performance after 1000 h of operation and found that Ni-YSZ showed better long-term stability, while Ni-GDC performed somewhat better in the short term [73]. They reported a lower overall degradation rate for Ni-YSZ (0.5 V% kh^−1^) than for Ni-GDC (0.7 V% kh^−1^) over the 1000 h test, a difference they linked to more stable microstructural behavior, less nickel agglomeration, and controlled TPB-length reduction in Ni-YSZ. Ni-GDC performed better initially but declined faster because the cell showed larger resistance changes from a higher initial ohmic resistance tied to a thicker electrolyte. Nenning et al. examined the relationship between microstructure, material properties, and electrochemical impedance in Ni-GDC cermet anodes. They reported low polarization resistance (below 0.1 Ω·cm^2^) and high power densities (2.4 W/cm^2^) at intermediate operating temperatures (600–700 °C), which they attributed to enhanced ionic and electronic conductivity arising from a distinctive microstructure produced by reductive sintering of Ni-GDC [74].

Scandia-stabilized zirconia (ScSZ) has drawn growing interest as an alternative to YSZ for SOFCs [75]. The two materials are broadly similar, though ScSZ shows a more complex phase structure and phase transitions. It is also more expensive than YSZ, largely because ZrO_2_ and Sc_2_O_3_, which make up 89–92 mol% of the composition, are in high demand and costly to produce [76]. ScSZ offers higher ionic conductivity and makes more oxygen ions available for carbon oxidation, and it also shows different carbon types and deposition behavior than Ni-YSZ owing to differences in crystal structure [77]. Arifin et al. examined the microstructural differences between YSZ and ScSZ and found that nickel interacts differently with the two materials, especially under reducing conditions. Ni particles paired with YSZ were finer after reduction than those paired with ScSZ. A related study in the same paper reported a weaker Ni-YSZ interaction (reduction temperature 377 °C), closer to that of pure NiO, versus a stronger Ni-ScSZ interaction (reduction temperature 507 °C), measured by temperature-programmed reduction (TPR). Ni-ScSZ remains susceptible to nickel coarsening, a common SOFC issue that reduces TPB area and affects anode durability and performance [76]. Arifin et al. compared Ni-ScSZ and Ni-YSZ under biogas operation and found that Ni-ScSZ performed better under these conditions, despite accumulating more carbon in the form of graphitic carbon [78]. The cell showed a smaller decrease in open-circuit voltage (OCV) and maximum power density when switching from hydrogen to biogas. The power density decreased by only 33.3% for Ni-ScSZ, compared with 86.5% for Ni-YSZ. Ni-ScSZ also showed a higher methane decomposition rate, supporting steadier hydrogen production that sustains performance even as carbon accumulates. The authors concluded that Ni-ScSZ better sustains SOFC operation under dry biogas because of this higher tolerance to carbon buildup.

Nickel-based composites with BaZrO_3_ (BZO), BaCeO_3_ (BCO), Y-doped BZO (BZY), Y-doped BCO (BCY) and Ba(Ce,Zr)O_3_ (BCZY) have emerged as key PCFC materials, each contributing distinct properties suited to specific application requirements. BZO offers excellent chemical stability, especially in atmospheres containing CO_2_ and H_2_O, but suffers from poor sinterability and grain-boundary conductivity [79]. In contrast, BCO shows higher proton conductivity and good sinterability but weaker chemical stability, which can lead to carbonate and hydroxide formation in reactive environments. Modified versions of BZO and BCO, namely BZY and BCY, build on the strengths of their parent materials. At an optimal Y-doping level (around 20 mol%), BZY shows enhanced conductivity without significantly compromising stability [80]. BCY offers strong proton conductivity and good performance at intermediate temperatures, although its long-term stability is limited by susceptibility to CO_2_ [79]. To address these limitations, researchers developed Y-doped Ba(Ce,Zr)O_3_ (BCZY), a composite that combines the high conductivity of BCO with the chemical stability of BZO.

A comparative study of Ni-BCZY, Ni-BCZY-GDC, and Ni-GDC anode films for PCFCs found Ni-BCZY gave the best combination of proton conductivity, anode resistance, and resistance to Ni grain growth after high-temperature sintering [81]. A separate study examined Y^3+^-doped Ba(Ce,Zr)O_3_ (BCZY) modified with activated carbon and combined with nickel oxide (NiO) for PCFC anodes, finding that the modification gave a finer, more porous microstructure and a lower ohmic resistance (R_0_ = 152 Ω·cm^2^) and polarization resistance (R_p_ = 46 Ω·cm^2^) than pristine NiO-BCZY (R_0_ = 161 Ω·cm^2^, R_p_ = 77 Ω·cm^2^) [82].

### 5.2. Nickel-Free Anodes

Nickel provides high catalytic activity for fuel conversion, but its susceptibility to carbon deposition and agglomeration has motivated the development of nickel-free alternatives. Rather than simply replacing Ni, these materials seek to retain sufficient catalytic activity while offering different combinations of electrical/ionic transport and surface reactivity. The following subsections examine the major nickel-free material classes and their compositional and structural characteristics, while their specific degradation mechanisms are discussed in Section 6.

One approach is to replace Ni with transition metals that have lower carbon-forming activity while maintaining adequate fuel-conversion kinetics. However, lower carbon-forming activity can also result in slower hydrocarbon activation, creating a trade-off between carbon tolerance and catalytic performance. This has encouraged the development of oxide-based materials, particularly ceria-based composites, perovskites, and spinels, whose catalytic properties can be tuned through composition, defect chemistry, and surface structure. Noble-metal systems such as ceria-supported Ru and GDC-supported Au can provide high catalytic activity but are constrained by their cost, motivating continued research into lower-cost nickel-free alternatives for hydrocarbon- and ammonia-fueled SOFCs.

#### 5.2.1. Ceria-Based Composites

To control carbon formation, commercial SOFC anodes can be modified with materials such as CeO_2_-based catalysts. Cerium (Ce), the most abundant rare-earth element, helps stabilize the catalyst by promoting carbon removal, owing to ceria’s high oxygen mobility. Ceria’s oxygen storage capacity (OSC) lets it release oxygen and offer an alternative route for oxidizing carbon particles even when ambient oxygen is scarce [83]. The electrochemical performance of cerium-based anode SOFCs is summarized in Table 2. Choolaei et al. found that a Co_1−x_-Zn_x_-Gd_0.1_Ce_0.9_O_1.95_ (x = 0.50 and 0.65) nanocrystalline anode powder performed well as a Ni-free, low-temperature SOFC (LT-SOFC) anode material [84]. The powder was synthesized by carboxylate co-precipitation, and the CoZn50-based cell reached a peak power density of 77 mW/cm^2^ at 750 °C, which the authors attributed to Zn addition improving performance at intermediate and low temperatures and raising the open-circuit voltage (OCV) to 0.91 V at 650 °C. Sarruf et al. investigated three anode types, CeO_2_-Co, CeO_2_-Cu, and a bimetallic CeO_2_-Co-Cu, and found that adding copper improved electrical conductivity and resisted coking, while cobalt improved catalytic activity [85]. Under hydrogen, methane, and ethanol fuels, the bimetallic anode reached notably higher power densities (538 mW/cm^2^ in hydrogen, 446 mW/cm^2^ in methane, and 395 mW/cm^2^ in ethanol at 850 °C) than the monometallic anodes, and it also showed strong resistance to carbon deposition during methane and ethanol operation, confirmed by Raman spectroscopy and temperature-programmed oxidation (TPO) analysis.

Doping ceria with cations such as Gd^3+^, Y^3+^, Pr^3+^, and Zr^4+^ can further raise oxygen mobility and OSC, and these dopants can also improve ceria’s ionic and electronic conductivity [86]. Because doping introduces mixed valences and oxygen vacancies into the unit cell, it alters both the physical and chemical properties of cerium, and with them, material stability [64]. Wang et al. reported that conductivity depends on the doping content and on the radius and charge of the dopant cation [87]. They also found that trivalent dopant cations (3+ charge) give higher ionic conductivity than di- or tetravalent dopants, and noted that dopant ionic radii close to that of cerium minimize structural distortion of the ceria lattice [88]. Bochentyn et al. identified Ce_0.9_Sm_0.1_O_2−δ_ and Ce_0.8_Pr_0.05_Sm_0.15_O_2−δ_ as particularly promising catalytic materials, obtained through aliovalent co-doping. In this approach, two dopant ions (Sm^3+^ and Pr^3+^) with valences different from that of the host cation (Ce^4+^) are introduced into the host lattice to create oxygen vacancies that preserve charge neutrality [89]. These vacancies enhance ionic conductivity, and the resulting materials showed the best long-term stability and carbon-deposition resistance of all the compositions tested.

**Table 2:** Summary of electrochemical performance of cerium-based anode SOFC materials.

No.	Anode	Dopant	Temperature (°C)	Fuel	Maximum Power Density (mW cm^−2^)	Reference
1	Cu-SDC	Sm	650	methanol	550	[90]
2	Cu-LaSDC	La	-	methanol	730	[90]
3	Cu-NdSDC	Nd	-	methanol	830	[90]
4	3CeO_2_-7CuO	Cu	550	hydrogen	587	[91]
5	Ru-SDC	Ru	450	methane	2.99	[92]
6	LSCF/GDC	Gd	750	hydrogen	1580	[93]
7	CoZn50	Zn	750	hydrogen	77	[84]
8	EDC15	Eu	550	hydrogen	-	[94]

#### 5.2.2. Perovskite-Based Anodes

**Figure 6:**
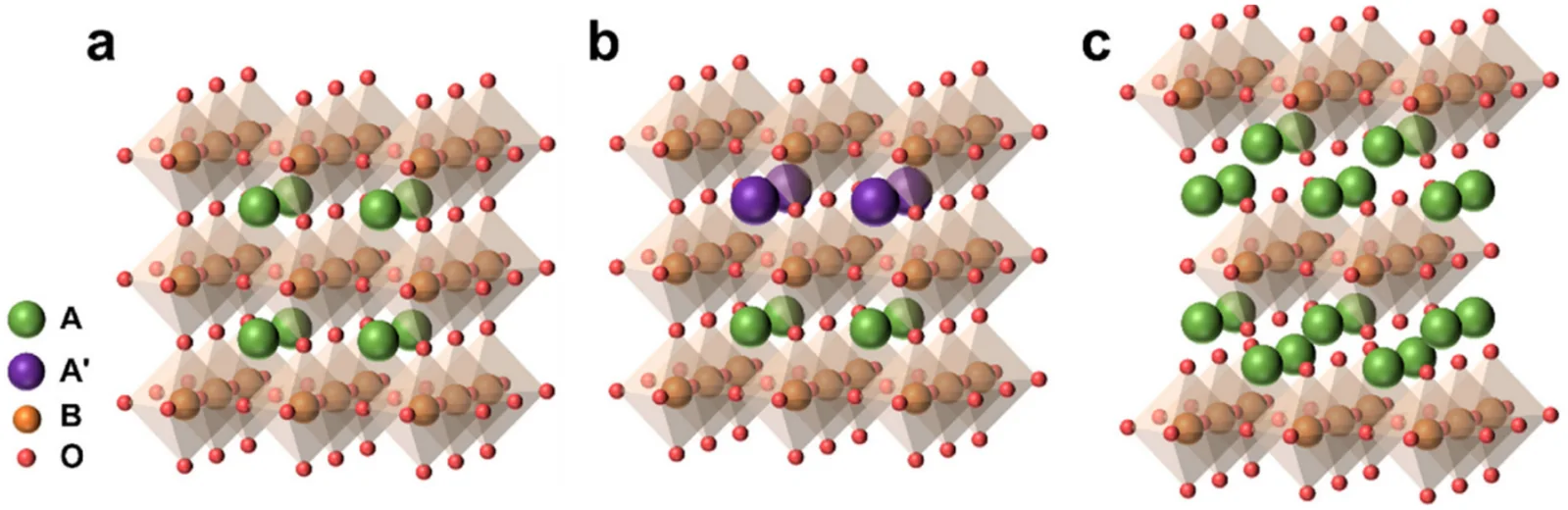
Schematic diagram of the three different types of perovskites: (**a**) cubic perovskites; (**b**) layered perovskites; and (**c**) Ruddlesden–Popper (RP). Reproduced from Ref. [97], © 2024 The Authors, licensed under CC BY 4.0.

Perovskite oxides are another class of anode material, with the typical formula ABO_3_, where A and B are cations whose combined charge totals +6 [95]. The A-site is usually occupied by a larger alkaline-earth, rare-earth, or lanthanide cation [96], while the B-site is typically occupied by a smaller transition-metal cation. The compositions of these sites and the concentration of oxygen vacancies influence oxygen-ion transport within the perovskite structure [97]. Perovskite oxides are known for mixed ionic-electronic conduction, sulfur-poisoning resistance, and, for some compositions, improved resistance to carbon deposition. As mixed ionic-electronic conductors, perovskite-based anodes can support oxygen-vacancy formation and oxygen transport, while their transition-metal sites can participate in hydrocarbon activation. Carbon buildup can be limited when oxygen species remain sufficiently mobile to oxidize carbonaceous intermediates [95]. Perovskites commonly adopt structures including simple cubic perovskite (ABO_3_), layered perovskites (AA′B_2_O_6_), and Ruddlesden–Popper phases (A_n+1_B_n_O_3n+1_) [97]. Figure 6 shows the crystal structures of these three perovskite types. Generally, structural differences among perovskite families affect oxygen-vacancy populations and oxygen-ion transport [97].

Oxygen transport through the perovskite lattice is a complex but essential process, occurring mainly by a vacancy mechanism in which oxygen ions hop between nearby oxygen vacancies. Three factors govern this transport: crystal structure, average metal-oxygen bond energy, and the concentration of oxygen vacancies. The main limitations of perovskite materials are their relatively low electrical conductivity and limited redox stability under operating conditions. Doping has become the primary strategy for addressing both, because the right dopant species allows researchers to modify the crystal structure, tailor the electronic structure, and raise the concentration of oxygen vacancies [18]. For instance, Rahumi et al. studied the double perovskite oxide Sr_2_Fe_1.5_Mo_0.5_O_6_ (SFM), known for its MIEC behavior, redox stability, and structural durability under SOFC operating conditions [98]. They introduced nickel (Ni) as a B-site dopant substituting for Fe, forming the doped compound Sr_1.9_Fe_1.4_Ni_0.1_Mo_0.5_O_6−δ_ (SFNM) to enhance electrocatalytic activity, improve electronic conductivity, facilitate oxygen-ion diffusion, and increase redox stability under fuel cell conditions. Among the tested compositions, SFNM2 (x = 0.2) performed best: the exsolved FeNi_3_ particles were well dispersed, providing a high, uniform surface area. This perovskite also showed low polarization resistance (R_p_) of 0.15 Ω cm^2^ at 800 °C, with a peak power density of 516 mW cm^−2^ at that same temperature under ammonia fuel. The cell reached an electrical efficiency of 39.7%, and long-term testing showed a low degradation rate of 0.48% per 100 h.

Another nickel-free perovskite anode, palladium-doped PrBaFe_2_O_5+δ_ (PBF), also shows strong durability and high performance under dry ethane, with excellent coking resistance during direct ethane oxidation [61]. Under reducing conditions, Pd@PBF undergoes in situ exsolution of Pd nanoparticles that anchor uniformly to the material surface, which markedly improves ethane gas adsorption and lowers the activation barrier from −0.14 eV in pristine PBF to −0.77 eV in Pd@PBF. The material also showed minimal degradation over 90 h of ethane operation, with peak power densities of 702 mW cm^−2^ when using H_2_ and 377 mW cm^−2^ under dry ethane at 800 °C [61]. In PCFC-specific work, a co-doped perovskite oxide, Ba_0.95_K_0.05_(Zr_0.1_Ce_0.7_Y_0.1_Yb_0.1_)_0.95_Mg_0.05_O_3*−*δ_ (BZCYYb-KMg), was studied, in which potassium (K^+^) and magnesium (Mg^2+^) were doped into the A-site and B-site of the BZCYYb structure, respectively [99]. According to the authors, this material markedly improved NH_3_ decomposition activity and proton conductivity, delivering roughly a 25% increase in power output over the undoped material. By raising NH_3_ decomposition activity and durability, K and Mg co-doping can improve the viability of ammonia-fueled PCFC anodes, although proton-conducting cells lie outside the main SOFC focus of this review.

#### 5.2.3. Spinel-Based Anodes

Spinel-type metal oxides, with the general formula AB_2_O_4_, have a cubic close-packed array of oxide ions with metal cations occupying tetrahedral (A-site) and octahedral (B-site) positions. Flores-Lasluisa et al. classify spinels as normal, inverse, or mixed, depending on cation distribution, with octahedral B-site cations playing the leading role in catalytic activity, particularly for the ORR and oxygen evolution reaction (OER) [100]. Spinel oxides are widely used in SOFCs because of their good thermal expansion compatibility with neighboring cell components, good electronic conductivity, and high chemical stability [101]. Their crystal structure lets cations occupy both the tetrahedral and octahedral sites, giving compositional flexibility and tunable electrochemical and mechanical properties [102].

Even though spinel oxides show encouraging structural and electrochemical properties, their application as Ni-free anodes is less established than their use as cathodes or interconnect coatings. For hydrocarbon-fueled SOFCs, their effectiveness relies on ensuring adequate electronic conductivity, catalytic activity, and phase stability in reducing environments. In addition, assessing the capability of spinel-based materials to withstand carbon buildup requires thorough analysis because variations in transition-metal oxidation states and phase composition may arise during fuel contact. Thus, additional experimental studies are required to clarify the relationships among spinel composition, stability in reducing atmosphere, and electrochemical performance in Ni-free anode applications.

#### 5.2.4. Role of Material Composition and Structure

Material composition and crystal structure determine the balance between catalytic activity, charge transport, oxygen mobility, and fuel tolerance in nickel-free anodes. Across ceria-, perovskite-, and spinel-based systems, cation substitution can modify oxygen-vacancy concentration, transition-metal valence, and surface reactivity, while composite structures can combine complementary ionic, electronic, and catalytic functions. For example, perovskite stoichiometry can strongly influence the electrochemical consequences of exsolution. Zhu et al. showed that introducing a 5% A-site deficiency in Sr(Ti,Fe,Ni)O_3−δ_ had little effect on the size and density of exsolved Fe–Ni nanoparticles, but helped maintain a more ordered perovskite structure after exsolution [103]. In contrast, the initially stoichiometric composition developed greater B-site deficiency, structural disorder, and strain after exsolution, resulting in substantially higher polarization resistance and poorer electrochemical performance. Similarly, exsolved metal–perovskite interfaces can provide active sites for hydrocarbon conversion while retaining the structural and transport functions of the oxide framework [104]. Experimental studies have demonstrated that controlled exsolution can enhance fuel-electrode kinetics and maintain structural stability under reducing conditions. As another example, Sun et al. demonstrated reversible exsolution of Bi and Fe nanoparticles from Bi-doped PrFeO_x−δ_, with XRD, TEM, and XPS confirming the formation and anchoring of the metallic phases [105]. The optimized electrode exhibited lower polarization resistance and higher power density than the undoped perovskite, reaching 0.63 W cm^−2^ with H_2_ and 0.47 W cm^−2^ with ethanol at 850 °C. These results support the role of exsolved metal–perovskite interfaces in enhancing fuel oxidation. However, because the hydrocarbon experiments in this study used ethanol rather than methane, methane-specific studies are still needed to confirm whether the same interfacial mechanism provides comparable coking resistance.

Ceria-based composites provide another approach in which the fluorite oxide supplies oxygen-ion transport and redox functionality, while a second phase provides catalytic activity. Spinels offer additional compositional flexibility because different cations can occupy tetrahedral and octahedral sites, allowing their redox and catalytic properties to be adjusted. Experimental work on Cu_1.3_Mn_1.7_O_4_ demonstrated that a spinel reforming layer could improve methane-fueled SOFC stability and carbon resistance through the formation of highly dispersed Cu during reduction [106]. These examples suggest that composition and structure should be designed together. Increasing catalytic activity alone is insufficient if the resulting phase loses transport pathways or promotes undesirable surface reactions. Table 3 summarizes representative composition–structure relationships for ceria-, perovskite-, and spinel-based materials, together with the fuels and operating conditions used to evaluate them.

## 6. Interaction of Ni-Free Anodes with Carbon-Based Fuels

This section discusses the interaction of Ni-free anodes with carbon-based fuels. Because many Ni-free oxides exhibit less facile hydrocarbon activation than metallic Ni, their principal advantage is not necessarily faster hydrocarbon conversion but a different reaction environment governed by surface and lattice oxygen, oxygen vacancies, and transition-metal redox chemistry. These characteristics can facilitate the oxidation and removal of carbonaceous intermediates and thereby reduce persistent carbon accumulation. For example, a Ni-free CeO_2_–Co–Cu anode demonstrated direct utilization of methane together with resistance to coke formation, showing that substantial hydrocarbon conversion can be achieved without relying on a conventional Ni-based anode [85]. Perovskite-based anodes are also considered promising alternatives because of their combination of redox stability, mixed ionic–electronic conductivity, compositional tunability, and resistance to carbon deposition. A representative Ni-free layered perovskite, (PrBa)_0.95_(Fe_0.9_Mo_0.1_)_2_O_5+δ_, demonstrated direct methane operation while maintaining high resistance to carbon build-up and sulfur poisoning, illustrating the potential of redox-active oxide anodes for direct hydrocarbon-fueled SOFCs [112]. Long-term operation has also demonstrated that this class of Ni-free oxide anodes can maintain stable performance under dry methane. A PBFM-based cell operated at 1 A cm^−2^ for 1060 h at 800 °C, highlighting the potential for combining hydrocarbon utilization with resistance to carbon deposition [112].

**Figure 7:**
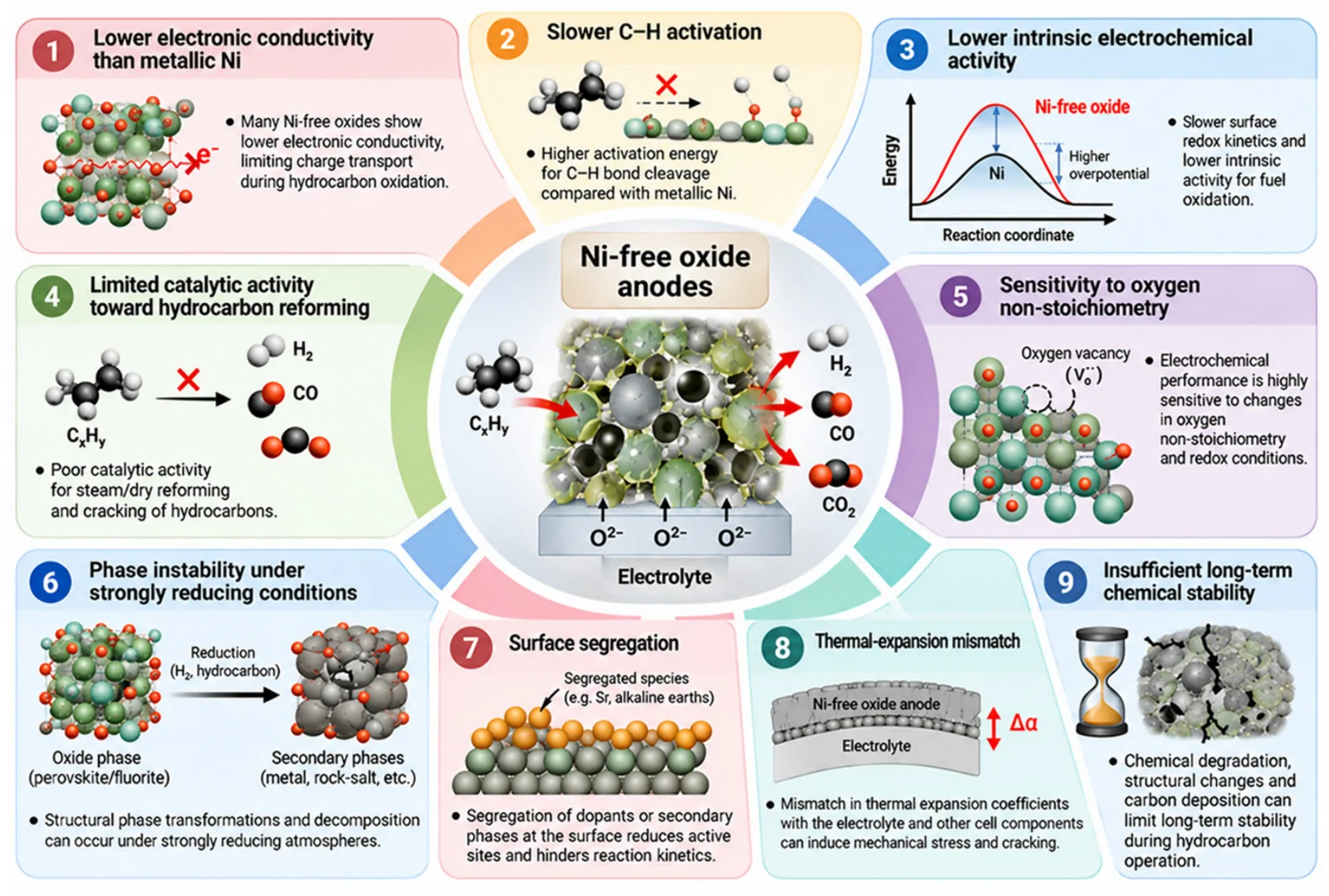
Key challenges and limitations of Ni-free oxide anodes for hydrocarbon-fueled SOFCs. Figure generated with the assistance of OpenAI ChatGPT (GPT-6).

However, these advantages come at a high cost, as Ni-free oxides can exhibit several limitations related to electrical conductivity, catalytic activity, oxygen non-stoichiometry, phase stability, surface chemistry, thermal compatibility, and long-term durability, as summarized in Figure 7. These limitations are important because carbon tolerance does not necessarily imply overall structural or electrochemical stability. For example, a recent study of SrFe_0.75_Mo_0.25_O_3−δ_/Ce_0.9_Gd_0.1_O_2−δ_ fuel electrodes showed promising initial performance but a higher polarization resistance than Ni/CGO, together with significant degradation caused by phase decomposition and microstructural reorganization during prolonged operation under hydrogen-rich conditions [113]. Thus, replacing Ni does not eliminate degradation but changes the dominant degradation mechanisms. The design objective therefore shifts from primarily controlling Ni coking and Ni oxidation toward controlling oxide redox chemistry, defect populations, surface reconstruction, phase evolution, and the stability of catalytic interfaces. These competing effects provide the basis for the following discussion of hydrocarbon activation, carbon formation and removal, and degradation mechanisms governing Ni-free anodes under carbon-containing fuels.

### 6.1. Hydrocarbon Activation and Carbon Formation

A generalized mechanism for methane conversion on a redox-active Ni-free oxide can be represented by the following sequence:

CH_4_(g) *→* CH*_4_ →* CH*_3_* + H* → CH_2_* + H* → CH* + H* (13)

followed by oxidation of carbonaceous intermediates:

CH*_x_* + O *→* CO* + H_x_O* (14)

CO → CO_2_* (15)

with concomitant regeneration of the oxide surface.

A parallel reforming pathway can proceed through hydroxyl species generated from steam:

H_2_O + V*_o_* → OH + H* (16)

followed by reaction of hydrocarbon fragments with surface hydroxyl groups. Such reactions can ultimately yield CO and H_2_ rather than fully oxidized CO_2_and H_2_O.

The overall surface chemistry can therefore be represented schematically as

CH_4_ → CH*_x_* → CH_x_O_y_
*→* CO* → CO_2_* (17)

or, under reforming conditions,

CH_4_ + H_2_O → CH*_x_* → CO + H_2_ → CO + H_2_* (18)

These pathways compete with carbon formation:

CH_4_ → C + 2H_2_* (19)

and

2CO ⇌ CO_2_ + C (20)

The key mechanistic variable is therefore not simply the intrinsic rate of hydrocarbon dissociation, but the relative rates of hydrocarbon activation and subsequent oxidation/removal of carbonaceous intermediates.

### 6.2. Carbon Formation and Removal on Ni-Free Anodes

Ni-free anodes encompass a broad range of oxide materials, including single and double perovskites, layered perovskites, Ruddlesden–Popper phases, SrTiO_3_-based oxides, fluorite-related ceria materials, spinels, and composite oxide architectures. Their interaction with hydrocarbon fuels differs fundamentally from that of conventional Ni-based cermets because hydrocarbon conversion is governed by oxide defect chemistry, transition-metal redox behavior, oxygen-ion transport, and the nature of the oxide surface rather than predominantly by metallic Ni sites. This distinction has motivated extensive development of Ni-free anodes for direct hydrocarbon SOFCs, particularly for methane, because lower carbon-forming activity can potentially be achieved without completely sacrificing fuel-conversion kinetics. However, experimental studies also demonstrate that Ni-free chemistry does not inherently eliminate carbon formation. Instead, it changes the balance between hydrocarbon activation, carbon generation, and carbon removal.

#### 6.2.1. Oxygen-Vacancy Chemistry and Carbon Removal

Oxygen non-stoichiometry is particularly important for reducible oxide anodes because oxygen vacancies influence oxygen transport, surface exchange, and the redox state of neighboring cations:

(21) $$O_{o}^{x}\text{ ⇌ }V_{\text{ₒ}}^{\text{••}}\;+\text{ ½}O_{2}(g)\;+\;2e^{\prime }$$

Several studies have shown that changes in oxygen-vacancy chemistry can influence the behavior of Ni-free anodes under hydrocarbon or alcohol fuels. Toscani et al. investigated two Ni-free ceria–zirconia compositions, Ce_0.9_Zr_0.1_O_2−δ_ and Ce_0.9_Zr_0.06_Sc_0.04_O_2−δ_. Sc substitution increased the defect-related features of the fluorite oxide and improved the behavior of the electrode under CH_4_ [114]. The Sc-containing composition showed lower polarization resistance and substantially lower carbon deposition than Ce_0.9_Zr_0.1_O_2−δ_. The authors related this improvement to changes in oxygen-vacancy concentration and mobility. However, the results also show that vacancy concentration alone cannot determine anode performance because oxygen transport, surface chemistry, and microstructure are closely coupled. Similar behavior has been reported for ceria-based Ni-free anodes containing Zr and Y.

Venâncio and de Miranda studied Cu-(Zr_x_Ce_1−x_Y_0.2_O_2−δ_-A_2_O_3_) Ni-free anodes for direct ethanol operation [115]. Their anodes contained ceria-based solid solutions with compositions of (Zr_0.25_Ce_0.75_)_0.8_Y_0.2_O_1.9_ and (Zr_0.50_Ce_0.50_)_0.8_Y_0.2_O_1.9_, together with an Al_2_O_3_ phase. The Zr substitution in the ceria lattice introduced structural defects and was associated with increased oxygen-ion mobility and changes in the Ce^4+^/Ce^3+^ redox couple. The Al_2_O_3_ phase also helped maintain thermal stability and limited coarsening of the ceria-based phase. These results show that oxygen-vacancy chemistry can improve oxygen storage and redox behavior, but its effect is also influenced by the surrounding phases and the stability of the composite structure. More recently, Wang et al. further demonstrated the importance of defect chemistry in the Ni-free layered perovskite PrBaFe_2_O_5+δ_ (PBF) and its high-entropy derivative La_0.2_Pr_0.2_Sm_0.2_Gd_0.2_Y_0.2_BaFe_2_O_5+δ_ (HEP) [116]. Replacing the Pr-site cation with five equimolar rare-earth elements disrupted the ordered structure and increased the oxygen-vacancy concentration. The HEP composition also showed improved hydration behavior, which promoted methane steam reforming and carbon removal. Under wet CH_4_ containing 3% H_2_O at 700 °C, the HEP anode maintained stable operation for 1000 h. Experimental Raman analysis also showed distinct carbon D- and G-bands at around 1350 and 1580 cm^−1^ on the PBF anode, while these bands were absent on the HEP anode. These results offer experimental evidence of enhanced resistance to detectable carbon buildup, whereas the suggested increase in carbon removal through improved hydration remains a mechanistic interpretation.

Taken together, these studies suggest that oxygen vacancies can promote carbon tolerance by supporting oxygen transport and the oxidation of hydrocarbon-derived species. However, a higher vacancy concentration should not be regarded as an independent measure of anode performance. The effect of vacancies depends on their concentration, mobility, interaction with the surrounding cations, and the ability of the oxide to maintain oxygen exchange under reducing fuel conditions. Therefore, effective oxygen-vacancy engineering requires a balance between defect formation, oxygen transport, surface reactivity, and structural stability.

#### 6.2.2. Hydrocarbon Activation Versus Carbon Accumulation

Carbon formation involves sequential hydrocarbon dehydrogenation followed by carbon nucleation and growth. For methane, this process can be represented as

CH_4_ → CH_3_ → CH_2_
*→* CH* → C* (22)

The subsequent fate of C* depends on the balance between carbon generation and its removal through oxidation or gasification. Therefore, carbon tolerance cannot be judged simply from the rate of hydrocarbon conversion. An anode can show high CH_4_ activation while maintaining low carbon accumulation if carbon removal is sufficiently fast.

This behavior was demonstrated by Sun et al. using La_0.4_Sr_0.5_Ba_0.1_TiO_3_ (LSBT) and a composite of LSBT with La_0.4_Ce_0.6_O_1.8_ (LSBT–LDC) [117]. The LSBT–LDC composite showed higher H_2_ formation and lower carbon deposition than LSBT during CH_4_ conversion. Quantitatively, they noted a catalyst weight increase of 6.1% for LSBT and 2.9% for LSBT-LDC after methane decomposition at 850 °C, which represents roughly a 52% decrease in weight gain for the composite catalyst. These experimentally measured values support the improved resistance to net carbon accumulation, while the proposed advancement in carbon removal by LDC remains a mechanistic interpretation. The results indicate that the addition of LDC did not simply suppress methane activation. Instead, it improved the balance between methane conversion and the removal of carbon formed during the reaction. During steam reforming, LSBT showed a gradual decrease in H_2_ production together with increased carbon formation, whereas LSBT–LDC maintained more stable H_2_ production with lower carbon accumulation [117]. The competing processes can be represented by

CH_4_(g) → C(s) + 2H_2_(g) (23)

C(s) + H_2_O → CO(g) + H_2_(g) (24)

These results show that improved coking resistance does not necessarily require a lower rate of hydrocarbon activation. A more useful criterion is whether carbon removal can keep pace with carbon formation. In the LSBT–LDC system, the LDC phase appears to promote both methane conversion and removal of carbon-containing species, resulting in lower net carbon accumulation [117].

A similar balance between fuel conversion and carbon tolerance has been reported for the A-site-deficient layered perovskite (PrBa)_0.95_(Fe_0.9_Nb_0.1_)_2_O_5+δ_ (PBFN). Ding et al. reported stable operation of PBFN in both humidified and dry CH_4_, with a peak power density of 0.57 W cm^−2^ in dry CH_4_ at 800 °C [118]. The material combines a layered perovskite structure with Nb substitution at the Fe site, which improves its chemical stability under reducing conditions. However, the study mainly demonstrated carbon tolerance through electrochemical performance and long-term stability rather than directly measuring the rates of carbon formation and carbon removal. Therefore, the results support stable CH_4_ operation but do not, by themselves, establish that enhanced carbon gasification is the dominant reason for the observed carbon tolerance.

A different situation occurs when a nominally Ni-free oxide undergoes exsolution. Zhang et al. investigated Sr_2_CoMo_0.95_Fe_0.05_O_6−δ_ (SCMF_0.05_), from which Co–Fe alloy nanoparticles were exsolved during reduction [119]. The exsolved Co–Fe nanoparticles increased CH_4_ oxidation activity, while the anode also showed strong coking resistance. The authors attributed this behavior to the combination of increased oxygen vacancies produced by Fe substitution and the catalytic activity of the Co–Fe nanoparticles for CH_4_ reforming to H_2_ and CO [119]. Post-test SEM and elemental mapping were performed after operation in wet CH_4_ to examine the carbon-resistant behavior of the SCMF_0.05_ anode. However, these characterizations do not provide a quantitative carbon deposition rate, and the proposed contributions of oxygen vacancies and exsolved Co–Fe nanoparticles to carbon removal remain mechanistic interpretations rather than directly measured carbon-removal kinetics.

The SCMF0.05 example also highlights an important limitation when interpreting the performance of exsolution-derived anodes. Exsolved metal nanoparticles can increase hydrocarbon activation while also providing sites where carbon formation may occur. Therefore, high CH_4_ conversion together with low carbon deposition should not automatically be attributed to oxygen vacancies alone. In SCMF0.05, the carbon-resistant behavior was associated with the combined effects of the exsolved Co–Fe phase and the surrounding oxide matrix. This distinction is important because the behavior of an exsolution-derived anode cannot be explained solely from the composition of its unreduced parent oxide. Exsolved metallic nanoparticles can improve hydrocarbon activation, but they could also serve as carbon nucleation sites, especially when hydrocarbon decomposition occurs faster than carbon oxidation or removal. This behavior is influenced by the composition of the metal, size of the particles, interactions at the metal-oxide interface, and the conditions of operation. In contrast to conventional supported metal nanoparticles, exsolved nanoparticles are frequently partially embedded within the oxide matrix, enhancing particle anchoring and decreasing vulnerability to carbon-induced detachment. However, exsolution by itself does not ensure total resistance to carbon deposits, and the enduring stability of these nanoparticles in hydrocarbon-rich environments needs additional investigation [120].

Future research must go beyond qualitative evaluations of carbon resistance by measuring the rates of carbon production and elimination under typical operating conditions. Carbon buildup can be assessed through thermogravimetric analysis (TGA), temperature-programmed oxidation (TPO), and carbon mass balance obtained from gas chromatography (GC). Where it is experimentally practical, the rates of carbon generation and elimination ought to be assessed independently under regulated fuel and oxidizing conditions. The net carbon accumulation rate, defined as the difference between carbon production and elimination rates, may act as a measurable indicator of coking vulnerability. Integrating these measurements with operando Raman spectroscopy and electrochemical degradation rates would yield a more thorough evaluation of the dynamic equilibrium between carbon deposition, carbon removal, and anode functionality [117].

Overall, these studies show that carbon tolerance in Ni-free anodes is controlled by the balance between hydrocarbon activation, carbon formation, and carbon removal. High fuel conversion is beneficial only when the resulting carbon-containing species can be removed at a sufficiently high rate. The composition of the oxide, the presence of secondary phases, oxygen-vacancy chemistry, and the formation of exsolved metal–oxide interfaces can all influence this balance.

### 6.3. Comparative Degradation Mechanisms

The improved carbon tolerance of Ni-free anodes does not imply immunity to degradation under carbon-based fuels. Instead, degradation results from the coupling of carbon formation with changes in surface chemistry, oxygen non-stoichiometry, mass transport, and electrode microstructure. The extent and dominant pathway depend on the oxide family, defect chemistry, fuel composition, steam content, temperature, and the structural changes that occur under reducing conditions.

**Figure 8:**
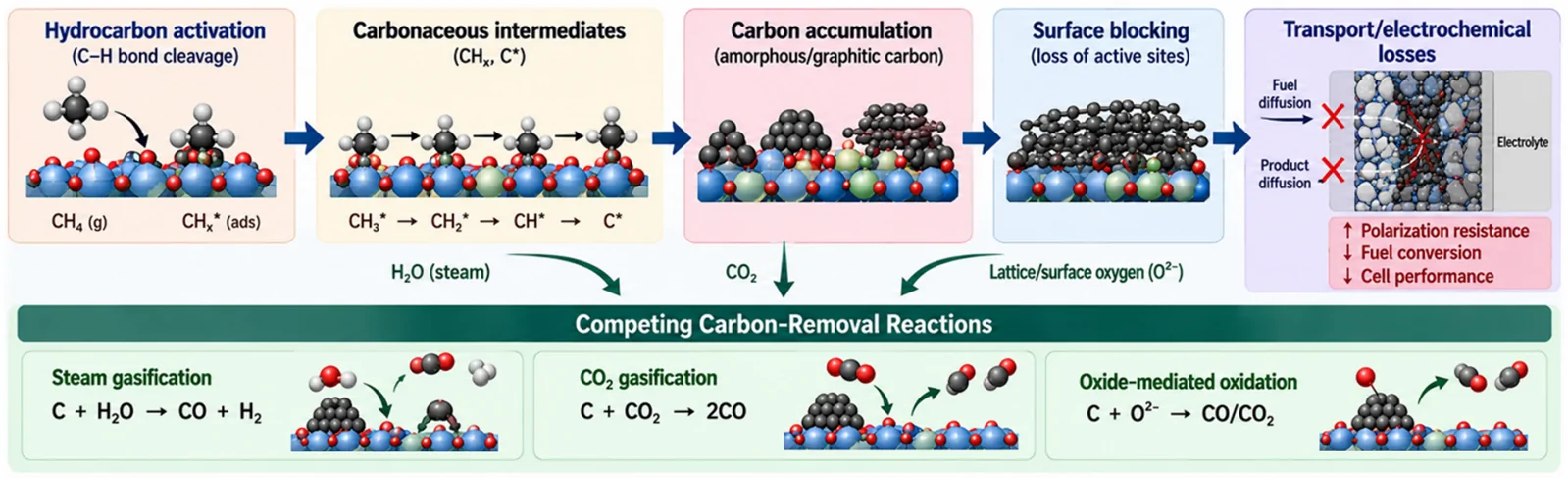
Schematic representation of the degradation pathway of Ni-free anodes under carbon-based fuels. Figure generated with the assistance of OpenAI ChatGPT (GPT-6).

A useful representation of the degradation sequence is shown in Figure 8, where hydrocarbon activation generates carbonaceous intermediates that can progressively accumulate on the surface. Continued carbon accumulation can block active sites and restrict fuel and product transport, ultimately increasing polarization losses and decreasing fuel-conversion efficiency. Carbon formation therefore represents only the initial stage of degradation. Its impact becomes more severe when deposited carbon is sufficiently persistent to modify the electrode surface or porous transport network.

The balance between carbon formation and removal is therefore central to determining the durability of Ni-free anodes. Carbon can be removed through steam (C + H_2_O → CO + H_2_) and CO_2_ gasification (C + CO_2_ → 2CO) as well as through reaction with reactive oxygen supplied by the oxide. The effectiveness of these pathways depends on oxygen-vacancy concentration, oxygen-ion mobility, surface redox chemistry, and the availability of steam or CO_2_. Thus, increasing the oxygen-vacancy concentration does not necessarily guarantee improved durability. Vacancies must remain sufficiently mobile and coupled to reversible oxygen exchange under the operating fuel conditions.

Experimental evidence illustrates this balance. Toscani et al. showed that Sc incorporation into CeO_2_–ZrO_2_-based Ni-free electrodes increased vacancy-related defect features and was associated with improved CH_4_ performance and lower carbon deposition [114]. However, the improvement cannot be attributed to oxygen-vacancy concentration alone because oxygen transport, surface exchange, and electrode microstructure also contribute to the observed behavior. Similarly, Sun et al. demonstrated that LSBT–LDC maintained higher H_2_ formation while accumulating less carbon than LSBT, indicating that improved carbon tolerance can result from more effective carbon removal rather than simply lower hydrocarbon activation [117]. These experimentally observed differences in carbon accumulation, as discussed in Section 6.2.2, support the importance of distinguishing measured carbon resistance from the proposed mechanisms responsible for carbon removal.

Degradation can also arise from structural evolution of the oxide itself. Under strongly reducing hydrocarbon atmospheres, transition-metal cations may change oxidation state, segregate to the surface, or undergo exsolution. These processes can initially improve catalytic activity by generating new active interfaces but may also alter the electrode structure during prolonged operation. For example, Wang et al. demonstrated that reduction of an LSCM-based composition generated embedded Cu–Fe nanoparticles and produced strong metal–oxide interfaces with good methane performance and resistance to filamentous carbon [121]. A key limitation is that exsolution changes the identity of the active surface. The resulting electrode is no longer simply the original oxide, and the exsolved metallic phase can both enhance hydrocarbon activation and introduce additional pathways for carbon growth.

**Figure 9:**
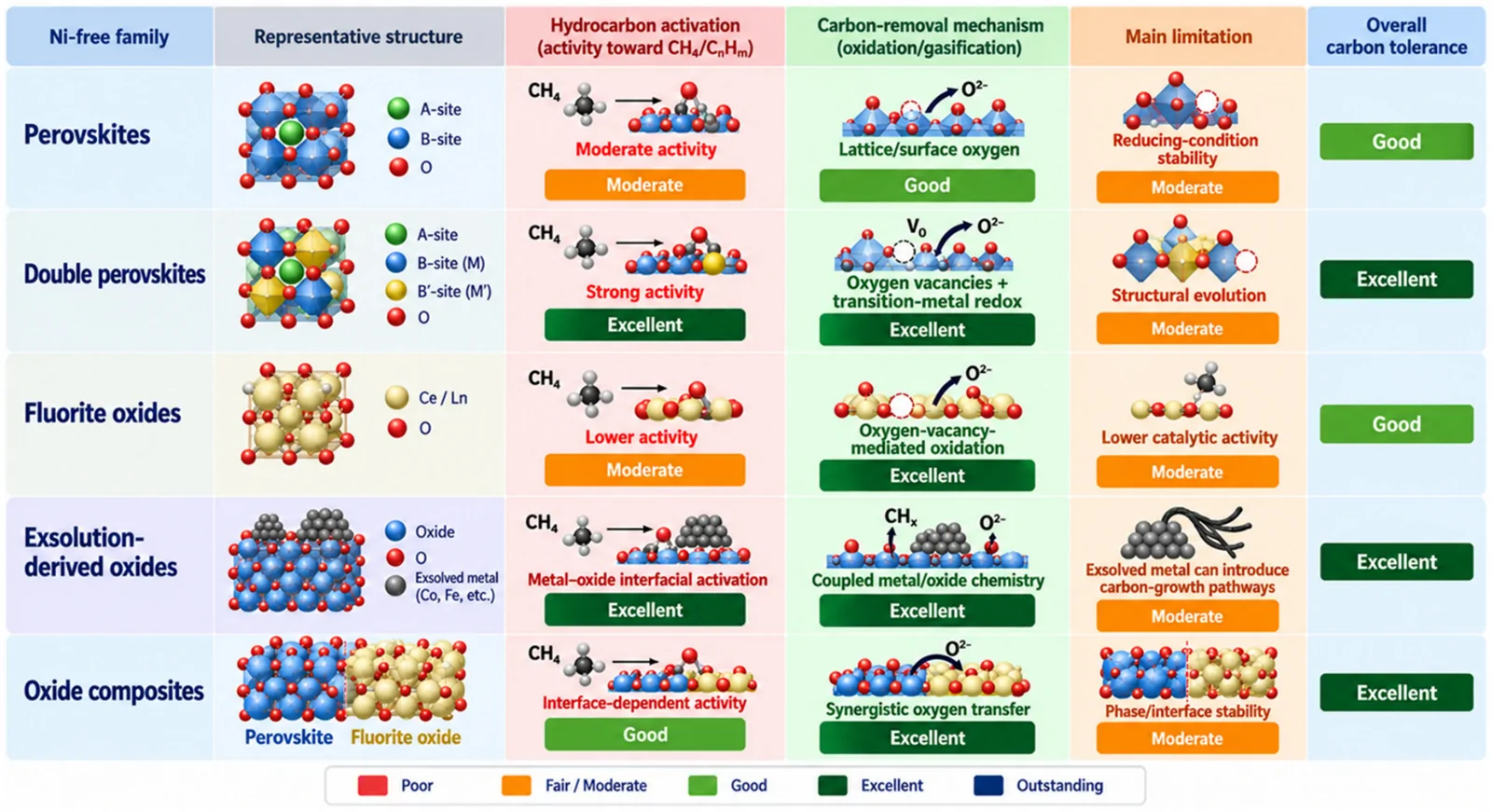
Qualitative comparison of Ni-free anode families for carbon-based fuel operation. Figure generated with the assistance of OpenAI ChatGPT (GPT-6).

Although exsolved metallic nanoparticles can improve fuel activation, their microstructural stability might be compromised during extended SOFC functioning. Possible degradation mechanisms encompass nanoparticle growth, surface restructuring, carbon buildup, and variations in metal-oxide interfacial interactions, which may lower the accessibility of active catalytic sites. Zhang et al. showed that extended reduction treatment facilitated the growth and coarsening of nanoparticles, resulting in a decline in electrochemical performance [119]. Nevertheless, these observations were gathered under regulated reduction conditions rather than during extended fuel-cell operation. Therefore, additional studies integrating prolonged electrochemical testing with microstructural analysis after operation are essential to determine how exsolved nanoparticles change and contribute to performance degradation under actual fuel conditions. These differences among Ni-free anode families are summarized in Figure 9. The comparison highlights that perovskites rely strongly on redox-active oxide surfaces and lattice and surface oxygen, double perovskites benefit from multiple transition-metal redox couples and oxygen-vacancy chemistry, fluorite oxides provide high oxygen storage and mobility, while exsolution-derived oxides introduce metal–oxide interfacial sites. Oxide composites can combine complementary properties through interfacial oxygen transfer. However, each architecture also introduces a distinct degradation concern, ranging from reducing-condition instability and structural evolution to lower hydrocarbon-activation rates or instability of exsolved metal–oxide interfaces.

An important limitation in assessing degradation is the frequent use of short-duration CH_4_ exposure as a measure of carbon tolerance. Low carbon accumulation after several hours does not necessarily demonstrate long-term structural stability. Longer-term operation can produce gradual cation redistribution, phase transformation, particle coarsening, or changes in oxygen non-stoichiometry that are not captured by initial coking tests. For example, Riegraf et al. reported significant degradation of SrFe_0.75_Mo_0.25_O_3−δ_/Ce_0.9_Gd_0.1_O_2−δ_ electrodes under H_2_-rich conditions that was associated with phase decomposition and microstructural reorganization rather than simply carbon deposition [113]. This indicates that a Ni-free electrode can exhibit limited coking while still undergoing substantial chemical or structural degradation.

Fuel composition provides an additional limitation. Carbon tolerance demonstrated with CH_4_ cannot automatically be extended to higher hydrocarbons. For example, Sun et al. detected carbonaceous species following ethanol operation of an exsolution-derived Bi–Fe-containing electrode, despite the favorable electrochemical behavior of the material [117]. This highlights the importance of evaluating Ni-free anodes using realistic carbon-containing fuels rather than relying exclusively on methane as a model fuel.

Overall, degradation under carbon-based fuels should therefore be considered as a coupled carbon–redox–transport problem. Carbon accumulation is one important pathway, but its impact is determined by the ability of the oxide to continuously supply reactive oxygen, remove carbonaceous intermediates, preserve its active surface, and maintain structural integrity under reducing conditions. The most convincing assessment of Ni-free anode durability requires simultaneous evaluation of carbon deposition, phase stability, surface chemistry, microstructure, electrochemical polarization, and long-term fuel-cell performance.

## 7. Challenges and Future Pathways

The development of SOFC anodes from conventional Ni-based cermets toward advanced oxide-based materials has significantly expanded the possibilities for fuel-flexible and impurity-tolerant operation. Nevertheless, achieving large-scale commercialization requires further progress beyond laboratory-scale demonstrations. In particular, the remaining challenges involve maintaining electrochemical performance under realistic fuel conditions, improving resistance to carbon- and sulfur-related degradation, ensuring redox and structural stability, reducing operating temperature, and developing manufacturing approaches that are scalable and economically viable. Future research should therefore move from isolated optimization of individual material properties toward integrated strategies that simultaneously address composition, defect chemistry, microstructure, interfaces, and long-term stability.

### 7.1. Material Design Strategies for Impurity-Resistant and Redox-Stable Anodes

Fuel impurities, particularly sulfur-containing compounds and carbon-forming species in hydrocarbon- and biogas-derived fuels, remain among the most persistent causes of anode degradation. Sulfur compounds can strongly interact with catalytic sites and inhibit fuel oxidation, while carbon accumulation can block pores and active surfaces, thereby increasing polarization losses. Therefore, the development of intrinsically impurity-resistant oxide anodes represents an important direction for future SOFC research. Several material-design strategies have shown promise. First, A-site deficiency and B-site substitution in perovskite oxides can modify oxygen mobility and surface chemistry, potentially reducing sulfur adsorption by maintaining a dynamic reservoir of surface oxygen [122]. Second, incorporating oxygen-storage materials such as doped ceria into composite anodes can enhance the electrochemical oxidation of carbon deposits and boost carbon tolerance [59]. Furthermore, exsolved perovskite-based catalysts have shown encouraging catalytic performance and fuel versatility, presenting an additional approach for creating degradation-resistant anodes [123]. Third, redox-stable mixed ionic–electronic conductors (MIECs) can help maintain electrical conductivity and catalytic activity during repeated transitions between oxidizing and reducing atmospheres.

These approaches also highlight an important limitation of composition-only optimization. Increasing oxygen mobility or redox activity may improve fuel conversion and impurity removal, but excessive reduction can destabilize the host structure, promote cation segregation, or trigger phase transformations. Therefore, future material design should aim not simply to maximize oxygen-vacancy concentration or catalytic activity, but to establish an appropriate balance between oxygen exchange, redox flexibility, structural stability, and impurity tolerance. Such an approach is particularly relevant for anodes intended for prolonged operation with carbon-containing and sulfur-containing fuels.

Future research needs to assess Ni-free anodes using realistic fuel compositions rather than relying only on pure or humidified laboratory fuels. For SOFCs fueled by hydrocarbons, testing must involve simulated biogas blends consisting of CH_4_ and CO_2_, along with controlled levels of H_2_S and other relevant impurities. For ammonia-fueled SOFCs, the influences of NH_3_ concentration, partial ammonia decomposition, and possible nitrogen-related deterioration need to be explored. These assessments must be performed at typical operating temperatures, fuel utilization rates, and current densities to determine whether the observed catalytic performance and impurity tolerance can be sustained in realistic operating environments [124].

### 7.2. Engineering Nanostructures and Interfaces for Lower Temperature Operation

Reducing the operating temperature of SOFCs is important for lowering degradation rates, reducing interfacial reactions and thermal stresses, and enabling greater use of lower-cost metallic interconnects and sealing materials. However, decreasing temperature also slows hydrocarbon conversion, surface exchange, ionic transport, and electrochemical reaction kinetics. This creates a need for electrode architectures that compensate for the kinetic limitations associated with lower-temperature operation without sacrificing long-term stability. Nanostructural and interfacial engineering therefore provide important routes for improving electrode performance. Hierarchical porous scaffolds can provide interconnected pathways for gas transport while increasing the accessible electrochemically active region [125]. Core–shell heterostructures provide another strategy by spatially separating functions within the electrode, for example by combining a highly conductive oxide core with a catalytically active surface layer [126]. Similarly, graded composite structures can distribute ionic and electronic conducting phases more effectively, reducing concentration polarization and improving reactant accessibility.

The benefits of nanostructuring, however, must be considered against the possibility of accelerated structural evolution. High surface area increases the number of active sites but can also increase the thermodynamic driving force for surface segregation, particle coarsening, and interfacial reactions during prolonged operation. Therefore, the future development of nanostructured anodes should focus not only on maximizing surface area but also on stabilizing the relevant interfaces under realistic fuel and redox conditions. Advanced fabrication techniques such as 3D printing, aerosol deposition, electrospinning, and freeze casting offer opportunities to control pore structure, phase distribution, and mechanical characteristics with greater precision. These approaches could facilitate the reproducible fabrication of hierarchical electrodes while providing improved control over transport pathways. At the same time, in situ and operando characterization techniques, including environmental TEM and synchrotron X-ray spectroscopy, will be increasingly important for identifying structural and chemical changes during actual operation and redox cycling [127]. Such information can help establish relationships between electrode architecture, dynamic surface evolution, and long-term degradation rather than relying solely on pre- and post-operation characterization.

Future durability evaluations ought to incorporate extended electrochemical testing under constant current density or fuel utilization settings, ideally for 1000 h or more where possible. Monitoring variations in cell voltage, area-specific resistance, and degradation rates is essential to assess long-term performance stability. Post-test XRD, SEM, and surface chemical analyses should complement these measurements to identify phase decomposition, nanoparticle coarsening, carbon buildup, and interfacial deterioration. These integrated electrochemical and microstructural analyses would yield a more dependable evaluation of anode durability than short-term performance assessments alone [128].

### 7.3. Engineering Reversible and Multi-Functional Anodes

The increasing integration of renewable energy into power systems places greater emphasis on flexible energy-conversion technologies capable of operating under changing electrical and fuel conditions. Reversible solid oxide cells (rSOCs), which can alternate between fuel-cell and electrolysis modes, provide a route toward integrating electricity generation, hydrogen production, and energy storage within a single platform [129]. For such applications, anode materials must withstand repeated changes between oxidizing and reducing environments while maintaining their structural integrity, electrical conductivity, and catalytic activity.

Perovskite-derived and spinel-like oxides containing robust oxygen sublattices are promising candidates because their structures can accommodate reversible oxygen incorporation and release without complete lattice collapse [130]. However, repeated redox cycling can also induce cation redistribution, phase transformation, particle growth, and changes in porosity. Therefore, the design of reversible anodes requires a careful balance between redox flexibility and structural rigidity. A material that exhibits high initial catalytic activity but undergoes irreversible reconstruction during cycling may be less suitable for long-term reversible operation than a moderately active material with greater structural stability.

Multifunctionality provides an additional design opportunity. Combining catalytic, ionic, and electronic functions within a single electrode architecture can reduce transport limitations and improve the utilization of active sites. Perovskite–ceria and perovskite–spinel combinations, for example, can provide complementary conductivity, oxygen-storage, and redox properties. Future research should further investigate the stabilization of catalytic nanoparticles, including Ru, Pd, or Co, within oxide structures to enhance electrochemical reactions during reversible operation. The broader objective is to develop electrode architectures in which catalytic activity, charge transport, oxygen exchange, and structural stability are integrated rather than optimized independently.

In addition to redox cycling, thermal cycling tests must be conducted to evaluate the mechanical and electrochemical stability of Ni-free anodes through repeated heating and cooling processes. These tests must include controlled temperature cycles between room temperature and the desired operating temperature, with cell voltage and polarization resistance monitored after each cycle under stable operating conditions. Microstructural characterization after cycling should be performed to detect any cracking, delamination of the electrodes, and degradation at the interfaces due to mismatches in thermal expansion among cell components. These tests are especially important for SOFC systems that experience frequent start-up and shut-down cycles [131].

### 7.4. Industrial Integration and Scalable Manufacturing

From an industrial and commercialization perspective, future progress must extend beyond material discovery to include scalable, reproducible, sustainable, and economically viable manufacturing. Laboratory-scale synthesis routes such as sol–gel, glycine–nitrate, and Pechini methods provide excellent control over composition and microstructure, but their translation to large-area electrode fabrication can be challenging. Greater emphasis should therefore be placed on adapting promising compositions to established manufacturing approaches such as tape casting, screen printing, and co-sintering while maintaining reproducibility in composition, porosity, and phase distribution.

The cost and availability of constituent elements are also important considerations. The development of Co- and Ni-free formulations is particularly attractive from both economic and environmental perspectives, encouraging greater investigation of Mn-, Fe-, and Cu-based perovskite and spinel systems. These more abundant elements can provide suitable redox and catalytic properties when their defect chemistry and electronic structure are appropriately controlled. However, reducing reliance on critical elements should not be pursued at the expense of long-term stability or manufacturing reliability. Material selection therefore needs to consider performance, resource availability, degradation behavior, and processing requirements simultaneously.

Environmental sustainability will become increasingly important as electrode production moves toward commercial scale. Future studies should incorporate life-cycle assessment (LCA) and green-chemistry principles to evaluate energy consumption, waste generation, and the environmental impact of precursor and electrode processing. Additive manufacturing and low-temperature sintering may further reduce processing energy requirements while improving resource efficiency. Ultimately, scalable manufacturing should be considered alongside material performance from the early stages of anode development rather than as a separate downstream challenge.

In addition to laboratory-scale testing, subsequent research must assess the scalability of Ni-free anodes by progressively validating from button cells to larger-area cells and multi-cell configurations. Significant focus must be placed on ensuring consistent electrode thickness, porosity, composition, and interfacial adhesion throughout large-scale manufacturing. The evaluation of electrochemical performance and degradation behavior must be conducted under typical stack operating conditions, incorporating uneven temperature distributions, fluctuations in fuel flow, and thermal stresses. Additionally, a systematic assessment of compatibility with electrolytes, interconnects, and sealing materials is essential to reduce interfacial reactions and mechanical failures. These evaluations are crucial for assessing whether promising laboratory-scale anode materials can deliver consistent performance and enduring reliability in real-world SOFC systems [132].

### 7.5. Summary and Outlook

The future development of SOFC anodes requires a transition from optimizing individual material properties toward integrated control across multiple length scales. At the atomic scale, composition and defect chemistry must provide appropriate oxygen mobility, redox flexibility, and resistance to fuel-derived impurities. At the microstructural scale, controlled porosity and stable interfaces are required to maintain efficient mass and charge transport. At the electrode and cell levels, these characteristics must be retained during prolonged operation, redox cycling, and exposure to realistic fuel compositions. Finally, scalable manufacturing and sustainable processing must be incorporated into material development to enable translation from laboratory-scale demonstrations to practical systems.

**Figure 10:**
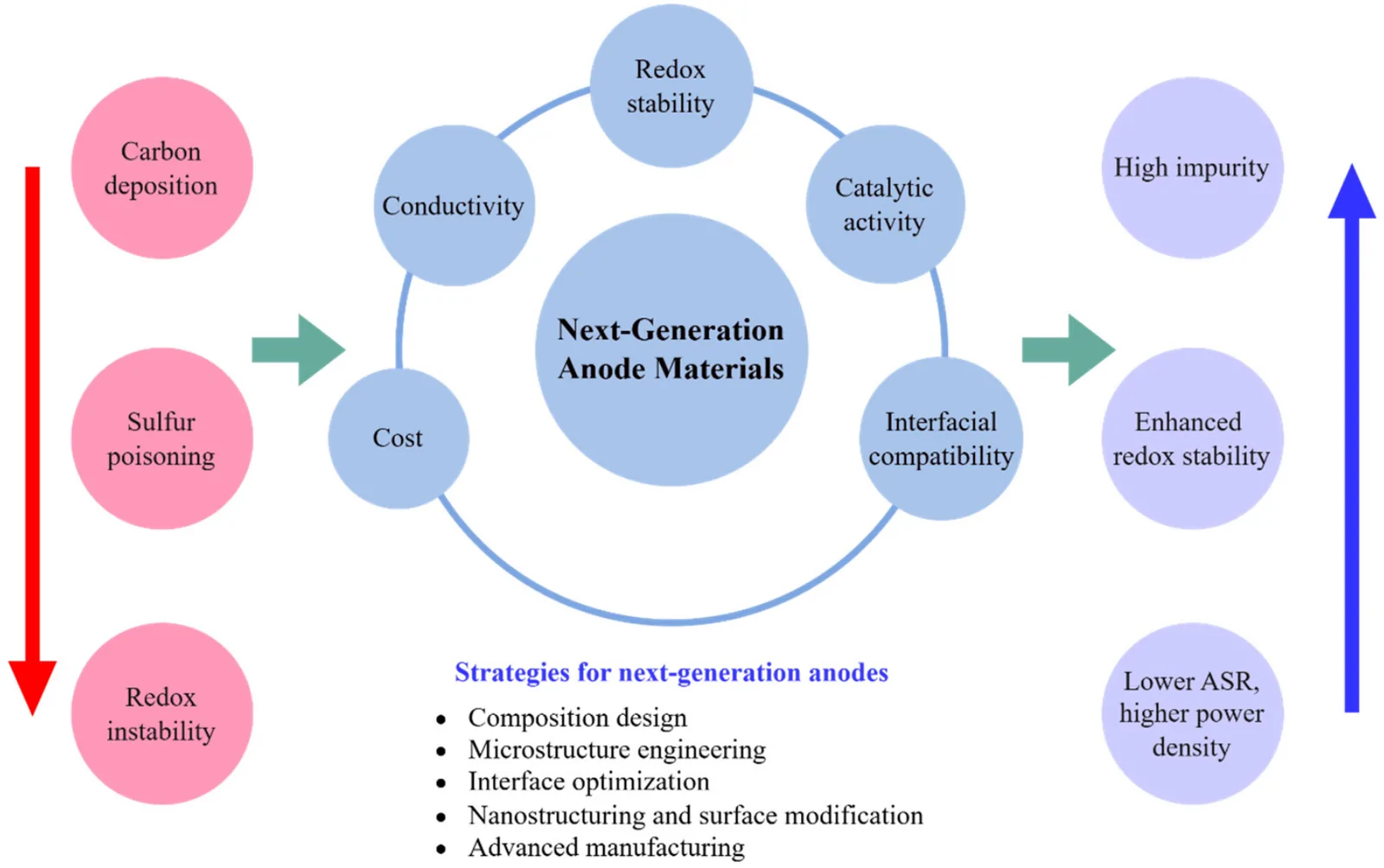
Schematic overview of the development strategies for next-generation SOFC anodes.

As summarized in Figure 10, the major challenges for next-generation SOFC anodes include carbon deposition, sulfur poisoning, redox instability, transport limitations, and the difficulty of maintaining performance during long-term operation. Addressing these challenges requires interconnected strategies involving composition and defect-chemistry control, microstructural engineering, interface stabilization, nanostructuring, reversible redox design, and advanced manufacturing. Rather than treating these strategies independently, future research should focus on identifying material architectures in which impurity tolerance, catalytic activity, charge transport, oxygen exchange, and structural stability reinforce one another. Such an integrated approach will be essential for developing fuel-flexible SOFC anodes that can maintain their performance under realistic operating conditions and ultimately support reliable and economically viable solid oxide energy-conversion systems. These interconnected strategies aim to improve impurity tolerance, maintain redox and structural stability, enhance electrochemical performance under realistic fuel conditions, and facilitate the transition of advanced SOFC anodes from laboratory-scale materials to practically manufacturable electrode systems.

## 8. Conclusions

This review shows that reliable SOFC operation with hydrocarbon and ammonia fuels requires anodes that combine sufficient fuel-oxidation activity with long-term stability. Nickel-free anodes provide an alternative to Ni-based systems by relying on oxygen vacancies, lattice oxygen, and transition-metal redox processes. However, they can still undergo phase transformation, cation segregation, exsolution, and microstructural changes during operation. High-temperature sintering during electrode manufacturing introduces an additional challenge, especially for porous composite oxides and nanoparticle-containing structures. High sintering temperatures or extended dwell periods can lead to nanoparticle growth, decrease the accessible catalytic surface area, and change the pore structure of the electrode. In addition, interdiffusion between the anode and electrolyte can form unwanted interfacial phases, which may increase polarization resistance and compromise long-term stability. Sintering temperature, dwell time, and electrode–electrolyte compatibility must therefore be carefully optimized. Alternative fabrication methods, such as low-temperature processing and post-sintering infiltration, should be considered to maintain active nanostructures and reduce interfacial reactions. The reviewed nickel-free anode families show different strengths. Perovskite and double-perovskite oxides offer flexibility in controlling redox behavior and oxygen vacancies, whereas fluorite oxides provide favorable oxygen storage and transport, and exsolution-derived materials and oxide composites can create active interfaces for fuel conversion and carbon removal. No single material family currently combines high activity, carbon tolerance, and long-term stability under all operating conditions. Under hydrocarbon fuels, performance depends strongly on the balance between hydrocarbon activation and carbon removal. Therefore, low carbon deposition alone does not guarantee long-term stability, as structural and microstructural changes may still occur. Future work should focus on long-duration testing under realistic fuels, more consistent testing protocols, control of defect chemistry and interfaces, and scalable fabrication. These efforts are essential for developing durable, high-performance nickel-free anodes for hydrocarbon- and ammonia-fueled SOFCs.

## Figures and Tables

**Table 1:** Main reactions of methane occur at the SOFC anode.

Reaction Type	Reaction Equation	SOFC Component	Remarks
Steam reforming (SR)	${\mathrm{CH}}_{4}+H_{2}O\;\text{→ }\mathrm{CO}+3H_{2}$	Anode	Endothermic reaction.
Dry reforming (DR)	${\mathrm{CH}}_{4}+{\mathrm{CO}}_{2}\;\text{→ }2\mathrm{CO}+2H_{2}$	Anode	Requires a nearly pure source of CO_2_ as feedstock. Utilizes CO_2_, thereby reducing greenhouse gas emissions.
Partial oxidation (POX)	${\mathrm{CH}}_{4}\;+\frac{1}{2}O_{2}\;\text{→ }\mathrm{CO}+2H_{2}$	Anode	Exothermic reaction.
Water–gas shift (WGS)	$\mathrm{CO}+{\;H}_{2}O\text{ →}\;CO_{2}+H_{2}$	Anode	Mildly exothermic. Increases hydrogen yield.
Electrochemical oxidation of hydrogen	$H_{2}+{\;O}^{2-}\;\text{→ }H_{2}O+2e^{-}$	Anode	Main electrochemical reaction in SOFCs.
Direct methane (DM) oxidation	${\mathrm{CH}}_{4}+O^{2-}\text{→ }{2H}_{2}+\mathrm{CO}+2e^{-}$	Anode	Bypasses reforming. Less common.
Complete oxidation (combustion)	${\mathrm{CH}}_{4}+2O_{2}\;\text{→ }{\mathrm{CO}}_{2}+2H_{2}O$	Anode	Overall chemical oxidation of methane. Highly exothermic.
Methane decomposition	${\mathrm{CH}}_{4}\text{ →}\;C+2H_{2}$	Anode	Produces carbon. Risk of carbon coking.

**Table 3:** Representative relationship between composition, structure, and function of nickel-free or Ni-reduced anode materials.

Material/Composition	Structure	Key Compositional/Structural Feature	Fuel/Atmosphere	Application	Main Functional Role	Key Experimental Observation	Reference
Doped CeO_2_ (GDC/SDC)	Fluorite	Dopant-induced oxygen vacancies	H_2_, CH_4_/reducing atmosphere	Anode/anode composite	Oxygen-ion transport and redox functionality	Ceria-containing systems provide oxygen mobility that can support hydrocarbon conversion and carbon removal.	[107]
La_0.2_Sr_0.8_Ti_0.55_Mn_0.35_X_0.1_O_3−δ_ (X = Ni, Co, Fe)	Perovskite	B-site substitution and oxygen non-stoichiometry	CH_4_/reducing atmosphere	Anode	MIEC transport and catalytic activity	B-site substitution and in-situ exsolution modified the catalytic metal–perovskite interface during methane operation.	[108]
Sr(Ti_0.3_Fe_0.63_Ni_0.07_)O_3−δ_	Perovskite	A-site stoichiometry controls exsolution	CH_4_/reducing atmosphere	Anode	Exsolved nanoparticle catalysis	A-site deficiency increased nanoparticle density, demonstrating the strong influence of bulk stoichiometry on surface structure.	[103]
Sr_1.9_Fe_1.5_Mo_0.5_Rh_0.2_O_6−δ_	Double perovskite	B-site Rh substitution and exsolved Rh–Fe alloy	CH_4_ + CO_2_	Anode	Dry-reforming and hydrocarbon conversion	Exsolved Rh–Fe/perovskite interfaces maintained stable methane–CO_2_ reforming at 800 °C for >200 h.	[109]
Cu_1.3_Mn_1.7_O_4_	Spinel	Cu/Mn mixed-valence framework; Cu reduction	CH_4_	Internal reforming layer/anode system	Hydrocarbon reforming	Reduction generated highly dispersed Cu and improved the stability of methane-fueled SOFC operation.	[106]
CuFe_2_O_4_	Spinel	Mixed Cu/Fe valence states	H_2_/reducing atmosphere	SOFC electrode	Catalytic/electrochemical activity	Demonstrated electrochemical activity, although direct evidence under hydrocarbon-fueled anode operation remains limited.	[110]
CoFe_2_O_4_	Inverse spinel	Co/Fe redox-active cation framework	CH_4_	Hydrocarbon catalyst/anode-related system	Hydrocarbon activation	Reduction generated active Co–Fe species, but carbon formation demonstrated the trade-off between catalytic activity and coking.	[111]

## Data Availability

No new data were created or analyzed in this study. Data sharing is not applicable to this article.
